# Development and validation of a new algorithm for improved cardiovascular risk prediction

**DOI:** 10.1038/s41591-024-02905-y

**Published:** 2024-04-18

**Authors:** Julia Hippisley-Cox, Carol A. C. Coupland, Mona Bafadhel, Richard E. K. Russell, Aziz Sheikh, Peter Brindle, Keith M. Channon

**Affiliations:** 1 Nuffield Department of Primary Health Care Sciences, University of Oxford, Oxford, UK; 2 Centre for Academic Primary Care, School of Medicine, University of Nottingham, Nottingham, UK; 3 King’s Centre for Lung Health, School of Immunology and Microbial Sciences, Faculty of Life Science and Medicine, King’s College London, London, UK; 4 Usher Institute, University of Edinburgh, Edinburgh, UK; 5 Population Health Sciences, University of Bristol, Bristol, UK; 6 British Heart Foundation Centre of Research Excellence, Radcliffe Department of Medicine, University of Oxford, Oxford, UK; 7 NIHR Oxford Biomedical Research Centre, Oxford University Hospitals NHS Foundation Trust, Oxford, UK

**Keywords:** Risk factors, Epidemiology

## Abstract

QRISK algorithms use data from millions of people to help clinicians identify individuals at high risk of cardiovascular disease (CVD). Here, we derive and externally validate a new algorithm, which we have named QR4, that incorporates novel risk factors to estimate 10-year CVD risk separately for men and women. Health data from 9.98 million and 6.79 million adults from the United Kingdom were used for derivation and validation of the algorithm, respectively. Cause-specific Cox models were used to develop models to predict CVD risk, and the performance of QR4 was compared with version 3 of QRISK, Systematic Coronary Risk Evaluation 2 (SCORE2) and atherosclerotic cardiovascular disease (ASCVD) risk scores. We identified seven novel risk factors in models for both men and women (brain cancer, lung cancer, Down syndrome, blood cancer, chronic obstructive pulmonary disease, oral cancer and learning disability) and two additional novel risk factors in women (pre-eclampsia and postnatal depression). On external validation, QR4 had a higher C statistic than QRISK3 in both women (0.835 (95% confidence interval (CI), 0.833–0.837) and 0.831 (95% CI, 0.829–0.832) for QR4 and QRISK3, respectively) and men (0.814 (95% CI, 0.812–0.816) and 0.812 (95% CI, 0.810–0.814) for QR4 and QRISK3, respectively). QR4 was also more accurate than the ASCVD and SCORE2 risk scores in both men and women. The QR4 risk score identifies new risk groups and provides superior CVD risk prediction in the United Kingdom compared with other international scoring systems for CVD risk.

## Abstract (web summary)

The QR4 algorithm for prediction of 10-year cardiovascular disease risk, developed, tested and externally validated in datasets comprising 16.8 million people from the United Kingdom, improves upon the QRISK3 algorithm that is in current use by incorporating new risk factors.

## Main

CVD is the leading cause of death globally and was responsible for an estimated 17.9 million deaths in 2019 (ref. ^1^). International guidelines from the World Health Organization^2^, United States^3^, Europe^4^ and the United Kingdom^5^ all recommend the use of CVD risk prediction tools to target those at high risk for interventions to reduce risk. Consequently, the effectiveness of public health policies relies on risk prediction tools that identify all the important risk groups in the population, with validated risk estimates across the full range of population characteristics. The United States recommends the ASCVD score, which is based on the Pooled Cohort Equations and was developed using 20,338 non-Hispanic and 1,647 African American individuals^3,6^. European guidelines recommend SCORE2, which was developed using data from 677,684 (refs. ^4,7^) and SCORE-OP^4^ developed using data from 28,503 participants from Norway^7^, and the United Kingdom recommends QRISK3, which was developed using a large, diverse community population of 7.9 million people^5,8^.

Recent research has highlighted conditions associated with increased CVD risk that are not captured by any of the three most widely used CVD equations globally: ASCVD (ref. ^3^), QRISK3 (ref. ^8^) and SCORE2 (ref. ^4,7^). These include chronic obstructive pulmonary disease (COPD)^9^, learning disability^10^, Down syndrome^11,12^, cancer^13^ and reproductive health conditions^14^. If these conditions are independently associated with increased CVD risk, then current CVD risk scores will underestimate risk in these groups, and people with these diagnoses may not be offered the opportunity for beneficial interventions to improve survival. Equally, if risks are overestimated, then individuals may receive unnecessary interventions^15^. Furthermore, more accurate CVD risk tools are useful for identifying those at higher CVD risk for recruitment into clinical trials, especially for primary prevention.

We sought to derive a new population-based CVD risk score, QR4, to include novel risk factors and account for competing risks, and to externally evaluate its performance against three widely used CVD risk scores (that is, ASCVD, QRISK3 and SCORE2), in large and diverse populations of over 16 million people drawn from across the United Kingdom. We used two established electronic records research databases (QResearch and Clinical Practice Research Datalink (CPRD) GOLD), both of which contain anonymized data collected during routine National Health Service (NHS) clinical care.

## Results

### Study population

There were 9,976,306 people aged 18–84 years in the QResearch English derivation cohort, 3,246,602 in the QResearch English validation cohort and 3,542,007 in the CPRD validation cohort from the other three UK nations (that is, Scotland, Wales and Northern Ireland). Extended Data Table 1 shows the flow of patients and the relevant exclusions. Extended Data Table 2 shows the new predictors under consideration.

The baseline characteristics of each cohort and the completeness of the recording of data for predictors with missing data are shown in Table 1. The cohorts were broadly similar, except that both English cohorts contained more complete data for ethnicity, smoking, cholesterol and body mass index (BMI) than the CPRD validation cohort from the other three UK nations. Supplementary Table 4 shows the characteristics of participants with complete versus missing data in the QResearch derivation cohort: those with complete data tended to be older, and more likely to be female and to have clinical conditions.

**Table 1:** Baseline characteristics of participants

	QResearch derivation cohort	QResearch validation cohort	CPRD validation cohort
Total	9,976,306	3,246,602	3,542,007
Men	4,820,711 (48.3)	1,564,545 (48.2)	1,698,728 (48.0)
Mean age (s.d.)	39.0 (15.0)	38.9 (14.9)	42.6 (16.4)
Mean Townsend (s.d.)^a^	0.7 (3.2)	0.9 (3.2)	0.0 (0.0)
Mean BMI (s.d.)	25.6 (5.2)	25.6 (5.2)	26.4 (5.0)
Mean cholesterol/HDL ratio	3.8 (1.2)	3.8 (1.2)	4.0 (1.3)
Mean SBP (s.d.)	123.6 (15.3)	123.4 (15.3)	125.4 (15.7)
Mean SBP variability (s.d.)^b^	9.3 (5.6)	9.3 (5.6)	9.6 (5.9)
Ethnicity recorded	6,186,167 (62.0)	1,972,052 (60.7)	1,257,906 (35.5)
White	4,391,142 (44.0)	1,392,310 (42.9)	1,155,924 (32.6)
Indian	301,414 (3.0)	95,018 (2.9)	19,217 (0.5)
Pakistani	186,029 (1.9)	50,470 (1.6)	11,116 (0.3)
Bangladeshi	115,682 (1.2)	42,898 (1.3)	3,884 (0.1)
Other Asian	218,555 (2.2)	67,456 (2.1)	10,776 (0.3)
Caribbean	103,578 (1.0)	34,397 (1.1)	1,272 (0.0)
Black African	285,326 (2.9)	94,302 (2.9)	14,653 (0.4)
Chinese	148,779 (1.5)	47,754 (1.5)	12,859 (0.4)
Other ethnicity	435,662 (4.4)	147,447 (4.5)	28,205 (0.8)
Smoking recorded	9,426,326 (94.5)	3,056,793 (94.2)	2,825,315 (79.8)
Nonsmoker	5,764,142 (57.8)	1,872,638 (57.7)	1,598,409 (45.1)
Ex-smoker	1,600,361 (16.0)	511,647 (15.8)	560,550 (15.8)
Light smoker (1–9 per day)	1,589,116 (15.9)	521,304 (16.1)	156,038 (4.4)
Moderate smoker (10–19 per day)	327,218 (3.3)	103,748 (3.2)	370,026 (10.4)
Heavy smoker (≥20 per day)	145,489 (1.5)	47,456 (1.5)	140,292 (4.0)
No learning disability	9,936,826 (99.6)	3,234,133 (99.6)	3,539,790 (99.9)
Other learning disability	34,663 (0.3)	10,962 (0.3)	335 (0.0)
Down syndrome	4,817 (0.1)	1,507 (0.1)	1,882 (0.1)
COPD	79,991 (0.8)	26,156 (0.8)	34,909 (1.0)
Lung cancer	4,422 (0.0)	1,353 (0.0)	1,786 (0.1)
Blood cancer	31,009 (0.3)	10,039 (0.3)	10,819 (0.3)
Brain cancer	1,245 (0.0)	370 (0.0)	381 (0.0)
Oral, lip, or throat cancer	3,864 (0.0)	1,220 (0.0)	1,427 (0.0)
Postnatal depression	96,463 (1.0)	29,763 (0.9)	32,468 (0.9)
Pre-eclampsia or eclampsia	20,233 (0.2)	6,735 (0.2)	8,637 (0.2)

Baseline characteristics are shown for individuals aged 18–84 years in the English QResearch derivation and validation cohorts and in the external CPRD validation cohort from Scotland, Wales and Northern Ireland. Participants were those without CVD and not on statins at study entry. Values are numbers (%) of participants, unless indicated otherwise. ‘White’ ethnicity includes British, English, Northern Irish, Scottish and Welsh.

^a^No practices in the CPRD validation cohort had Townsend deprivation scores because these data were unavailable, so we assumed a value of zero.

^b^Based on an s.d. of two or more values.

There were 202,424 incident CVD cases (based on primary outcome definition) from 49.1 million person-years in the derivation cohort. Extended Data Table 1 shows the types of CVD events in each cohort for each of the three outcome definitions.

**Extended Data Fig. 1:**
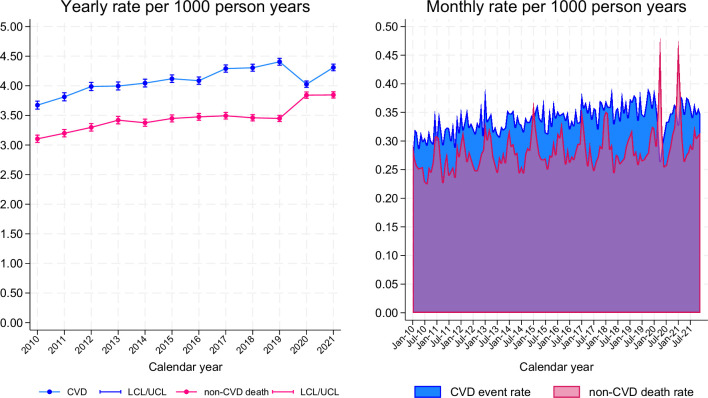
CVD (primary outcome) and non-CVD death rates per 1000 by calendar year and month over the full study period in the English derivation cohort in those aged 18–84 years. The two red spikes on the monthly graph show the non-CVD deaths occurring during the first and second COVID-19 pandemic waves in April 2020 and Jan 2021. Data presented in left panel are mean rates with 95% confidence intervals.

The crude CVD incidence rates for the primary CVD outcome by age, sex, ethnicity and calendar year in the English derivation cohort and CPRD validation cohort are shown in Extended Data Table 3. CVD rates using linked data were higher in the English cohort, which was largely explained by the additional data linkage to hospital and mortality data. Extended Data Fig. 1 shows both CVD incidence rates and non-CVD mortality rates by calendar year and month for the whole study period. CVD rates per 1,000 person-years were lower in 2020, the first year of the COVID-19 pandemic, when the overall rate was 4.03 (95% CI, 3.97–4.08) but returned to pre-pandemic levels in 2021 (4.31; 95% CI, 4.25–4.37). Non-CVD mortality rates increased from 3.45 (95% CI, 3.40–3.50) in 2019 to 3.84 (95% CI, 3.79–3.89) in 2020 and remained elevated in 2021.

### Factors associated with increased risk of CVD

**Fig. 1:**
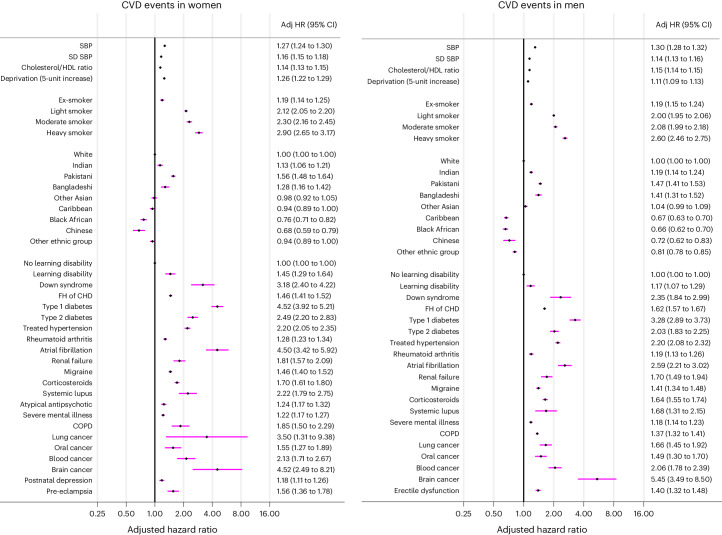
Final model-adjusted hazard ratios for CVD. Adjusted hazard ratios in 5,155,595 women and 4,820,711 men, presented at the mean age of 39 years for variables with age interactions. The hazard ratios were adjusted for fractional polynomial terms for age and BMI (see Supplementary Fig. 1, which shows the relevant fractional polynomial terms). SBP is per 20-unit increase. Adj HR, adjusted hazard ratio; FH of CHD, family history of coronary heart disease.

**Extended Data Fig. 2:**
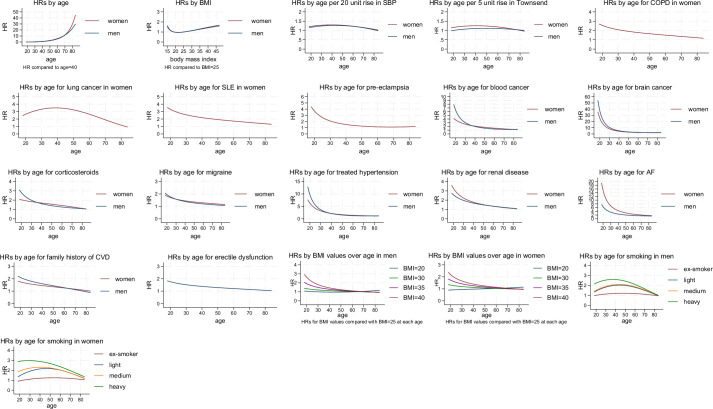
Adjusted hazard ratios for CVD risk for fractional polynomial terms for age and BMI and age interactions in the derivation cohort. Fractional polynomial terms for adjusted hazard ratios for CVD risk for age, BMI and age interactions in the derivation cohort.

The adjusted hazard ratios for CVD incidence in the final cause-specific models in men and women (evaluated at the mean age of 39 years for variables with age interactions) are shown in Fig. 1. Extended Data Fig. 2 shows the adjusted hazard ratios for the fractional polynomial terms for CVD risk for continuous variables and the predictor variables with significant age interactions for both men and women. Supplementary Figs. 1 and 2 show the corresponding results for non-CVD death.

There were seven new CVD predictors in men and women (brain cancer, lung cancer, Down syndrome, blood cancer, COPD, oral cancer and learning disability) and two additional predictors in women (pre-eclampsia and postnatal depression).

We found no association between the following variables and CVD risk in men or women: asthma, hyperthyroidism, hypothyroidism, antiphospholipid antibody syndrome, benign intracranial hypertension, HIV or AIDS, and the remaining cancers. In women, there were no associations with in vitro fertilization, endometriosis, polycystic ovarian syndrome, gestational diabetes, miscarriage, termination or placental abruption. No violations of the proportional hazard assumptions were detected graphically. The values for the heuristic shrinkage^16^ were all very close to one (0.99), indicating no evidence of overfitting.

#### New CVD predictors in women

The adjusted hazard ratios (95% CI) for the nine new independent predictors of CVD risk in women (evaluated at the mean age of 39 years for variables with age interactions) are as follows: brain cancer, 4.52 (2.49–8.21); lung cancer, 3.50 (1.31–9.38); Down syndrome, 3.18 (2.40–4.22); blood cancer, 2.13 (1.71–2.67); COPD, 1.85 (1.50–2.29); oral cancer, 1.55 (1.27–1.89); learning disability, 1.45 (1.29–1.64); pre-eclampsia, 1.56 (1.36–1.78); and postnatal depression, 1.18 (1.11–1.26).

The adjusted hazard ratios for several of these predictors were higher at younger ages (for example, under 35 years), except for lung cancer in women, for which adjusted hazard ratios were highest for those around age 40 years and then declined gradually with increasing age (Extended Data Fig. 2). The adjusted hazard ratios (95% CI) at age 69 were as follows: brain cancer, 2.18 (1.29–3.71); lung cancer, 1.97 (1.64–2.37); blood cancer, 1.39 (1.28–1.50); COPD, 1.38 (1.32–1.44); and pre-eclampsia, 1.12 (1.01–1.24).

The magnitude and direction for many of the adjusted hazard ratios for the competing outcome of non-CVD death in women were similar to those for CVD except for large adjusted hazard ratios (evaluated at age 39 years) for Down syndrome (18.32; 95% CI, 16.24–20.66), lung cancer (49.94; 95% CI, 40.61–61.43) and brain cancer (33.35; 95% CI, 26.17–42.49). The adjusted hazard ratios for non-CVD death for family history of coronary heart disease, pre-eclampsia and migraine were significantly less than one (Supplementary Fig. 1).

#### New CVD predictors in men

The adjusted hazard ratios for the seven new independent predictors of CVD risk in men (evaluated at age 39 years are shown in Fig. 1), and the adjusted hazard ratios (95% CI) for these predictors are as follows: brain cancer, 5.45 (3.49–8.50); Down syndrome, 2.35 (1.84–2.99); blood cancer, 2.06 (1.78–2.39); lung cancer, 1.66 (1.45–1.92); oral cancer, 1.49 (1.30–1.70); COPD, 1.37 (1.32–1.41); and learning disability, 1.17 (1.07–1.29). The adjusted hazard ratios in men for brain cancer and blood cancer declined with age; for example, at age 69 years, the adjusted hazard ratio (95% CI) was 2.12 (1.25–3.61) for brain cancer and 1.23 (1.15–1.31) for blood cancer.

The adjusted hazard ratios for the additional models were similar to our final main models in men and women (Supplementary Figs. 3–7). Model A includes the original QRISK3 predictor variables but without competing risks. Model B is similar to our final model, but the follow-up time ended on 29 February 2020, before the COVID-19 pandemic. Model C shows that the adjusted hazard ratios for CVD risk were similar across periods of time after diagnosis with one of the four cancers (except for oral cancer in women), although the adjusted hazard ratios for non-CVD deaths varied with the highest values for more recently diagnosed cancers.

### Predicted risks

**Fig. 2:**
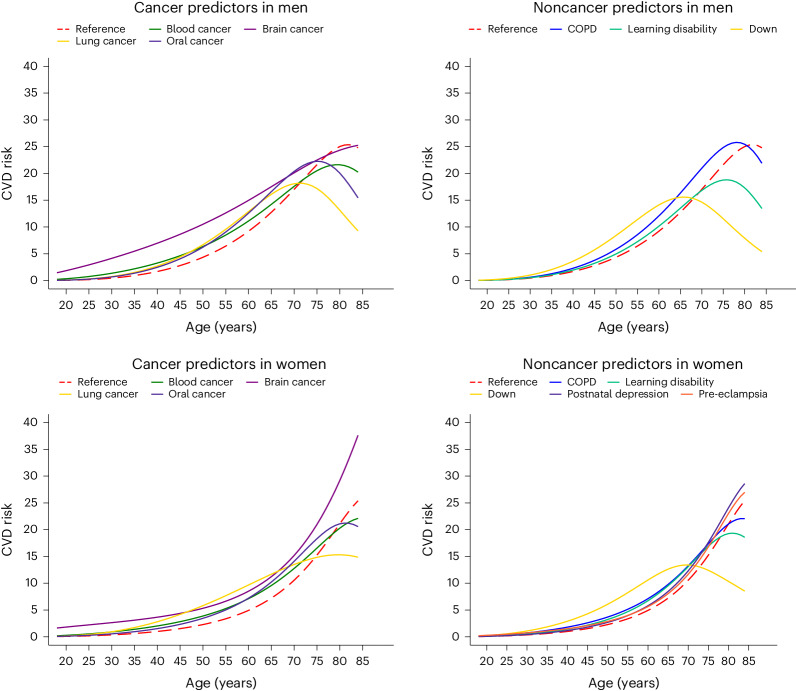
Effect of the new risk factors on prediction of 10-year CVD absolute risk. Ten-year CVD risk predictions for men and women over different ages. Predictions for an individual with each of the new risk factors are compared to those of a similar individual of the same age but without the new risk factors (reference individual). In this analysis, the reference individual is a White nonsmoker and has no adverse health conditions, an SBP of 125 mm Hg, a cholesterol/HDL ratio of 4.0 and a BMI of 25 kg m^−2^.

The way in which each of the new risk factors affects the predicted 10-year CVD risk for specific individuals is shown in Fig. 2. In this illustration, which is presented for both men and women across ages 18 to 84 years, CVD risk was compared between individuals with a new risk factor and reference individuals with no adverse clinical indicators (a cholesterol/HDL ratio of 4.0, an SBP of 125 mm Hg and a BMI of 25 kg m^−2^). These risk calculations show the impact of the new risk predictors, which mainly resulted in increased predicted risks compared with the reference individuals at younger ages and decreased predicted risks at older ages as competing risks become more pronounced. Using a reference group of individuals with various conventional risk factors (light smokers with a cholesterol/HDL ratio of 6.0, an SBP of 170 mm Hg and a BMI of 35 kg m^−2^), a similar pattern was observed, albeit with higher overall predicted risks (Supplementary Fig. 8).

### Discrimination

The performance statistics (C statistic, calibration slope and calibration intercept) for QR4 and QRISK3 for the validation cohorts in England, Scotland, Wales and Northern Ireland are shown in Table 2. The C statistic for QR4 was marginally higher than that for QRISK3 in both validation cohorts. For example, the C statistics were 0.835 (95% CI, 0.833–0.837) and 0.831 (95% CI, 0.829–0.832) for QR4 and QRISK3, respectively, in women in the devolved administrations (Scotland, Wales and Northern Ireland). The corresponding values in women in England were 0.864 (95% CI, 0.862–0.866) for QR4 and 0.862 (95% CI, 0.860–0.864) for QRISK3. The C statistic values were generally higher in England than in the other three nations, although all values remained within an excellent range (>0.8). The results for men were similar, though the values were slightly lower.

**Table 2:** Evaluation of discrimination and calibration of QR4 compared with QRISK3

	Women		Men	
	QRISK3, mean (95% CI)	QR4, mean (95% CI)	QRISK3, mean (95% CI)	QR4, mean (95% CI)
**England**				
C statistic	0.862 (0.860 to 0.864)	0.864 (0.862 to 0.866)	0.848 (0.846 to 0.850)	0.849 (0.847 to 0.851)
Calibration slope	1.00 (0.994 to 1.01)	0.870 (0.863 to 0.878)	1.01 (1.01 to 1.02)	0.900 (0.894 to 0.907)
Intercept	0.003 (−0.006 to 0.013)	−0.130 (−0.137 to −0.122)	0.0136 (0.006 to 0.022)	−0.100 (−0.106 to −0.093)
**Devolved administrations**				
C statistic	0.831 (0.829 to 0.832)	0.835 (0.833 to 0.837)	0.812 (0.81 to 0.814)	0.814 (0.812 to 0.816)
Calibration slope	1.68 (1.66 to 1.69)	1.21 (1.2 to 1.22)	1.61 (1.6 to 1.62)	1.24 (1.23 to 1.25)
Intercept	0.676 (0.662 to 0.69)	0.211 (0.204 to 0.219)	0.608 (0.597 to 0.62)	0.238 (0.231 to 0.245)
**Wales**				
C statistic	0.823 (0.82 to 0.827)	0.829 (0.825 to 0.832)	0.809 (0.806 to 0.812)	0.812 (0.809 to 0.815)
Calibration slope	2.07 (2.04 to 2.11)	1.35 (1.34 to 1.37)	2.06 (2.03 to 2.09)	1.40 (1.39 to 1.42)
Intercept	1.07 (1.04 to 1.11)	0.353 (0.338 to 0.368)	1.06 (1.03 to 1.09)	0.405 (0.391 to 0.418)
**Scotland**				
C Statistic	0.833 (0.83 to 0.835)	0.837 (0.834 to 0.839)	0.813 (0.811 to 0.815)	0.815 (0.812 to 0.817)
Calibration slope	1.5 (1.48 to 1.51)	1.14 (1.13 to 1.15)	1.44 (1.43 to 1.46)	1.16 (1.15 to 1.17)
Intercept	0.496 (0.48 to 0.512)	0.136 (0.126 to 0.145)	0.444 (0.431 to 0.457)	0.162 (0.154 to 0.171)
**Northern Ireland**				
C statistic	0.844 (0.838 to 0.85)	0.847 (0.841 to 0.853)	0.821 (0.817 to 0.826)	0.823 (0.818 to 0.828)
Calibration slope	1.53 (1.49 to 1.58)	1.15 (1.13 to 1.18)	1.29 (1.26 to 1.32)	1.09 (1.06 to 1.11)
Intercept	0.535 (0.49 to 0.58)	0.153 (0.127 to 0.179)	0.292 (0.262 to 0.321)	0.0855 (0.0644 to 0.107)

The discrimination and calibration of QR4 were compared with those of QRISK3 in people aged 18–84 years in the internal QResearch (England) and external CPRD (devolved administrations) validation cohorts on the basis of the primary outcome measure.

The overall discrimination results and discrimination results delineated by ethnic group for QR4, SCORE and ASCVD are shown in Extended Data Table 4; these results were restricted to those aged 40 years and older in the validation cohort in England. For women, overall discrimination was highest with QR4 (0.781; 95% CI, 0.778–0.784), followed by ASCVD (0.767; 95% CI, 0.764–0.770) and SCORE2 (0.767; 95% CI, 0.764–0.770). There was a similar pattern for men.

The C statistics, calibration slopes and calibration intercepts (overall and by ethnic group) for QRISK3 and QR4 in men and women aged 18–84 years in the English validation cohort are shown in Extended Data Table 5. These results show that discrimination varied by ethnic group in England: the C statistic for QR4 was highest for Chinese men (0.923; 95% CI, 0.906–0.939) and lowest for Caribbean men (0.825; 95% CI, 0.801–0.841).

The definitions of the CVD outcomes used for sensitivity analyses are shown in Supplementary Table 1. Supplementary Table 2 shows the performance statistics for QR4, SCORE2 and ASCVD for each outcome measure for each of the four UK nations among those aged 40 years and older. The C statistic values for all scores (QR4, SCORE2 and ASCVD) were highest for the tertiary outcome measure, and for all outcome measures discrimination values were higher for QR4 than those for SCORE2 and ASCVD, which yielded values similar to one another.

### Decision curve analysis

**Fig. 3:**
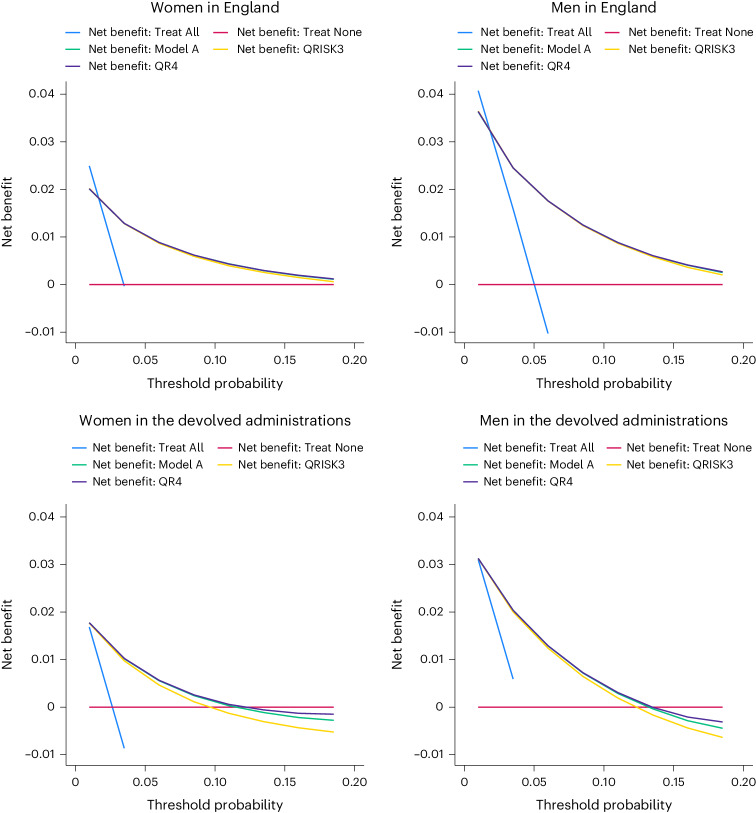
Decision curves for QR4, QRISK3 and Model A. Decision curves showing net benefit in men and women aged 18–84 years in England and the devolved administrations. Decision curves for QR4, QRISK3 and Model A are compared to those for ‘Treat All’ (intervention in all individuals irrespective of risk threshold) and ‘Treat None’ (intervention in no individuals).

The decision curves in Fig. 3 indicate a slightly larger net benefit with QR4 compared with QRISK3 and Model A, but differences in net benefit are more marked in the devolved administrations than in England.

**Extended Data Fig. 3:**
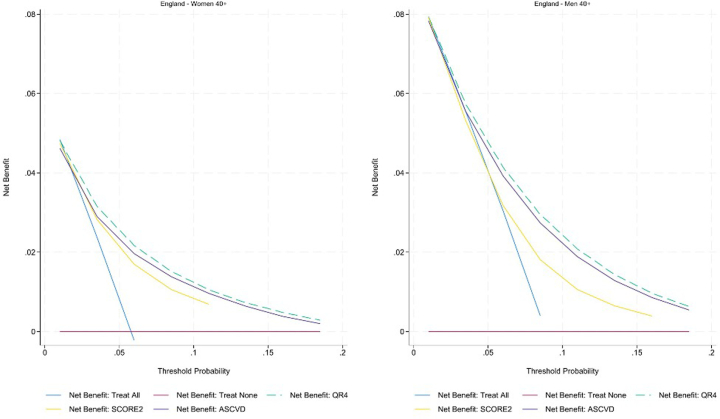
Decision curves for QR4, ASCVD and SCORE2 in people aged 40+ in the England validation cohort using the primary CVD outcome definition. The greatest net benefit is observed for QR4 followed by ASCVD followed by SCORE2.

The decision analysis curves for QR4, SCORE2 and ASCVD for the primary outcome in England are shown in Extended Data Fig. 3. Supplementary Figs. 9 and 10 show corresponding results for the secondary and tertiary CVD outcomes.

### Calibration

**Fig. 4:**
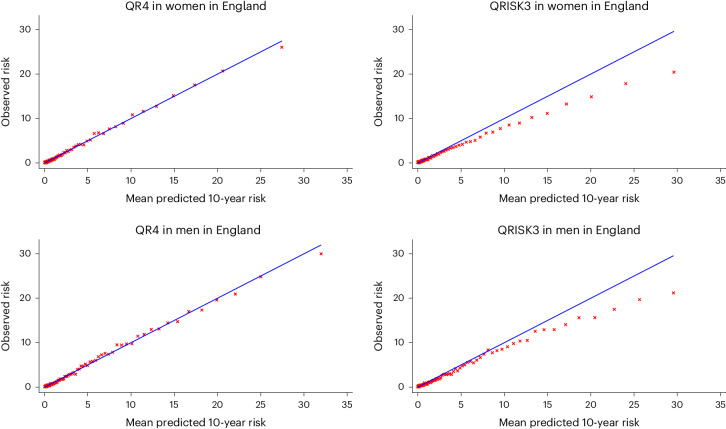
Calibration of QRISK3 and QR4. Centile calibration plots of the observed and predicted risks for QR4 and QRISK3 in men and women aged 18–84 years in the English validation cohort. The red crosses show the observed risk versus the 10-year risk of CVD at each level of mean predicted risk. The blue line shows a perfect calibration scenario in which the mean predicted risk is equal to the observed risk.

QR4 was well-calibrated in England, showing a close correspondence between predicted and observed risks, whereas QRISK3 overpredicted risk in the higher centiles of predicted risk (Fig. 4). Table 2 shows the calibration slope and intercept values for QRISK3 and QR4 by country. There was a degree of miscalibration for QRISK3 and QR4 in each of the devolved administrations (Supplementary Fig. 11) on the basis of general practitioner (GP) data only.

**Extended Data Fig. 4:**
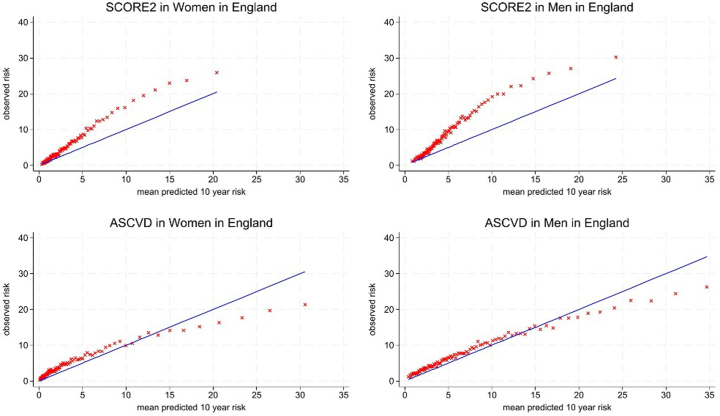
Predicted and observed 10-year CVD risks for ASCVD and SCORE2 in the English validation cohort in people aged 40+ using the primary CVD outcome. Predicted and observed 10-year CVD risks for ASCVD and SCORE2 in the cohorts from England in people aged 40+ using the primary CVD outcome definition.

The calibration results for ASCVD and SCORE2 in the English validation cohort, which are based on our primary outcome definition, are shown in Extended Data Fig. 4. Supplementary Table 2 and Supplementary Figs. 12 and 13 show the corresponding results for the secondary and tertiary outcomes. Overall, there was a degree of overprediction for ASCVD and a degree of underprediction for SCORE2, which were improved when comparisons were made with the more specific secondary and tertiary outcomes.

### Reclassification

The characteristics of the 84,700 (2.6%) participants in the English validation cohort reclassified using QR4 instead of QRISK3 at the 10% risk threshold are shown in Extended Data Table 6. Of the 3,554 people reclassified from low risk to high risk using QR4, 1,168 (32.9%) had COPD, 57 (1.6%) had a learning disability, 72 (2.0%) had Down syndrome, 72 (2.0%) had a history of pre-eclampsia, 125 (3.5%) had a history of postnatal depression, 90 (2.5%) had oral cancer, 54 (1.5%) had brain cancer, 92 (2.6%) had lung cancer and 322 (9.1%) had blood cancer. Those reclassified as high risk using QR4 tended to be younger (mean age of 52.4 years) than the 81,146 people reclassified as low risk (mean age of 60.5 years). Supplementary Table 3 shows the corresponding analyses for the 4,068 participants reclassified as high risk using QR4 compared with Model A as well as the 12,791 participants reclassified as low risk; the pattern was similar, although the total number of participants that were reclassified was much smaller (16,859, 0.52%).

## Discussion

We have developed and externally validated a new CVD risk score, QR4, that incorporates nine novel predictors with good face validity and clinical utility to predict 10-year risk of CVD in a diverse population of men and women. These new predictors (in both men and women) are learning disability, Down syndrome, COPD, lung cancer, oral cancer, blood cancer and brain cancer, and pre-eclampsia and postnatal depression in women only. The performance of QR4 was more accurate than other widely used CVD risk scores, namely, ASCVD, QRISK3 and SCORE2. QR4 is likely to result in clinically important changes in risk, leading to different CVD risk reduction advice or interventions, particularly for those with the new predictors, which might lead to interventions at an earlier age, as in the examples given. Furthermore, QR4 accounts for the competing risk of non-CVD death, thereby reducing overprediction of risk, especially among the more elderly populations^17^. Last, we have used and published the SNOMED-CT clinical code groups used to derive our model, facilitating reuse for further research and international comparisons.

Widely available CVD risk equations have been used for many millions of CVD health checks worldwide and are supported by international guidelines^2–5^. However, it is important that guidance is based on the best algorithms available because this will materially affect which patients are offered risk-reducing interventions. Failure to adequately assess CVD risk and offer appropriate risk-reducing interventions across all patient groups could further disadvantage vulnerable patients, particularly cancer survivors and those with significant comorbidities such as COPD, Down syndrome, a learning disability or a history of postnatal depression or pre-eclampsia. Although the underlying conditions themselves may not be modifiable, the identification of high-risk people in these groups can lead to targeted interventions to reduce CVD risk.

Our findings regarding QR4 for cancer are particularly striking and confirm associations with CVD risk for four cancers (that is, blood, brain, lung and oral)^13^ despite accounting for reduced life expectancy using a competing risk analysis. The increased risk of CVD for cancer survivors particularly at younger ages needs to be considered in the context of the prognosis of the cancer itself because it would be inappropriate to prescribe therapies that lower CVD risk for those with a very poor prognosis. Although only 15% of people with lung cancer survive more than 5 years, 90% of people with blood cancers^18^ and 55% of people with oral cancers now survive more than 5 years^19^, and hence, targeted prevention has a potential clinical net benefit. The use of QR4 in clinical practice will need careful consideration and discussion in patients with cancer and will need to account for patient preferences. There are also opportunities for further research to more finely characterize the association between cancer treatment(s) and subsequent CVD. However, longitudinal data on cancer treatments (such as radiotherapy and chemotherapy) are only just becoming available for this type of research data in the United Kingdom. These data are not yet routinely linked to primary care data for clinical use, so at present, they could not be used to implement more personalized risk prediction.

The lack of an association between asthma and CVD risk is interesting, especially given the preconception that inhaled corticosteroids may increase the risk of CVD. By contrast, the 1.4-fold to 1.9-fold increased risk of CVD associated with COPD is consistent with the 2-fold increased risk of CVD reported among US patients hospitalized with COPD^9^ and is clinically very important. COPD is now one of the top three most deadly diseases worldwide, resulting in an estimated 3 million deaths annually, 90% of which occur in low-income and middle-income countries^20^. It is striking that this association was strongest in women with COPD, and there are two important implications of this finding. First, clinicians need to actively consider COPD as a diagnosis and to confirm it with spirometry, especially in women who are often neglected in this regard^21,22^. Second, therapies that reduce CVD risk should be prescribed; these therapies include optimizing inhaled therapies, as this has now been demonstrated to reduce mortality^23,24^.

The increased risk of CVD associated with pre-eclampsia declined with age, but the 54% increase that we observed at a mean age of 39 years is consistent with other research^25,26^ and may reflect damage to the maternal cardiovascular system^26^. This highlights an important opportunity to systematically target CVD prevention^14^. The twofold to threefold increased risk of CVD among those with Down syndrome is consistent with the results of the limited analyses available^11^ and may reflect premature aging and adverse cardiometabolic profiles. This underscores US recommendations for continued CVD research and surveillance in people with Down syndrome, especially given improved life expectancy^12^. Incorporation of postnatal depression and learning disabilities into QR4 will help to operationalize policy initiatives to ensure parity of esteem with physical health for these patients.

Our study reports robust discrimination for ASCVD and SCORE2. Although there was a degree of miscalibration with ASCVD and SCORE2 with the main outcome, which used a broader definition of CVD, this improved with endpoint definitions aligned with those for which ASCVD and SCORE2 were developed. Any residual miscalibration compared with the original studies may relate to a combination of different study populations (which might have different underlying CVD rates), different cohort selection criteria (for example, the inclusion of statin users in the SCORE2 studies), use of a different study period, and use of recalibration measures in SCORE2 (including the differential application of multipliers by age and sex, which are yet to be validated). This suggests that CVD risk equations may be transportable to other geographical settings if recalibrated.

The strengths and limitations of this study are similar to those for other well-established risk prediction tools. The strengths include size, duration of follow up, representativeness, lack of selection, recall and respondent bias, and no evidence of overfitting^8^. The inclusion of more granular information on predictors is a strength in that the predictions for individual patients are likely to better reflect their individual risk, although this needs to be balanced against the increased complexity of the algorithm with regard to its implementation. However, this is mitigated in settings for which electronic health records are available because most relevant information is already available at the point of care and can be automatically populated^27^. Although we report improved discrimination for QR4 compared with QRISK3, the absolute values of the improvement in the C statistics were small. The C statistic is a familiar but limited measure that does not effectively balance misclassification errors^28^. It needs to be interpreted in the context of other relevant measures, including decision analysis, reclassification, calibration and clinical utility^28^. Our study has strong face validity because it was conducted in a setting where most patients are managed, and hence, QR4 could be implemented in similar clinical settings, subject to local validation or recalibration. Last, our results are unlikely to have been affected by the COVID-19 pandemic in 2020 and 2021, as the risk factors were predominantly recorded prior to the pandemic. The CVD incidence rates were temporarily affected in 2020 but have since returned to pre-pandemic levels, and Model C showed very similar results to our main model.

One limitation is the lack of formal adjudication of CVD diagnoses. However, the use of linked hospital and mortality data ensures a clear ascertainment of CVD outcomes in the English cohorts. The miscalibration for QR4 in the other three UK nations (Scotland, Wales and Northern Ireland) reflects the lack of linked hospital data and Office for National Statistics mortality outcome data for these nations in the CPRD validation cohort, as this would have resulted in an underestimation of CVD outcomes. Although there is potential for bias because of missing data, our data are substantially more complete than previous studies^8^, with the mitigation of residual biases through multiple imputations using recommended approaches^29^. We expect that in clinical practice, any missing data will be collected from the patient or their caregiver during a consultation with the clinician, so missing data for the implementation of QR4 are unlikely to be a substantial issue. Although our validation covers a fully external population, further research should validate QR4 in different countries with different CVD rates. This could be addressed by further validation using different datasets with appropriate data linkages.

In conclusion, these results demonstrate the strength of QR4 in the general UK population and its superior performance compared with three other widely used international CVD risk scores. QR4 enables more accurate CVD risk estimation, which should lead to significant improvements in health outcomes, especially for those with COPD, Down syndrome and a learning disability, cancer survivors and women with pre-eclampsia or postnatal depression.

## Methods

### Study design and data sources

We undertook community-based cohort studies using two large electronic medical records databases, QResearch and CPRD GOLD. We randomly allocated three-quarters of QResearch practices in England to the derivation cohort and the remainder to an internal English validation dataset. Both QResearch and CPRD GOLD are based on anonymized medical records data collected during the course of clinical care. QResearch is based on a commercial computer system known as Egton Medical Information Systems (EMIS), whereas CPRD GOLD is based on a different commercial system known as Vision. We used CPRD GOLD practices from three UK nations (Scotland, Wales and Northern Ireland) to create a second fully external geographically distinct validation cohort.

We included adults aged 18–84 years between 1 January 2010 and 31 December 2021. The cohort entry date was the latest of the following: 18th birthday, date of registration with the practice plus 1 year or 1 January 2010. We excluded participants with pre-existing CVD, those prescribed statins and (for QResearch) those with a missing Townsend deprivation score because they often represent temporary or incompletely registered patients with substantial missing data^30^. We followed participants up until the earliest date of CVD diagnosis, death, deregistration with the practice or the study end date.

### Outcome definitions

Our primary outcome for model derivation and validation in QResearch was an incident diagnosis of CVD (fatal or nonfatal myocardial infarction, ischemic heart disease, ischemic, hemorrhagic or unspecified stroke or transient ischemic attack) identified from the GP record or linked mortality and hospital records using published clinical codes^31^. Our primary outcome for model validation in CPRD GOLD was based on the same diagnoses but recorded solely from the GP data because linked data for deaths and hospital admissions were not available for Scotland, Wales and Northern Ireland.

We had two additional outcomes for the validation comparisons between QR4, SCORE2 and ASCVD. Our secondary outcome, aligned with ASCVD, included nonfatal myocardial infarction or coronary heart disease-related death and fatal or nonfatal stroke. Our tertiary outcome was similar to our secondary outcome but also included fatal congestive cardiac failure, hypertension and cardiac arrhythmias in order to align with the SCORE2 outcome definition^4,7^. For more details of the definitions of the primary and two additional outcomes, including the SNOMED-CT and ICD-10 codes used, see Supplementary Table 1. We compared the performance of all three algorithms (QR4, SCORE2 and ASCVD) using all three outcome definitions only in England because of the availability of linked cause of death data, which were not available for the devolved administrations.

### Predictor variables

We included established risk factors from ASCVD^3^, QRISK3 (ref. ^8^) or SCORE2 (ref. ^4^) and new candidate variables highlighted in the literature (see Extended Data Table 2, which includes more details of the definitions of each predictor considered)^9,10,11,12,13,32^. Cholesterol ratio was defined as total serum cholesterol/HDL. Ethnicity was self-reported.

### Model development

We used cause-specific Cox models to estimate the 10-year risk of CVD, accounting for non-CVD death as a competing risk for men and women separately using the biological sex recorded on the electronic health record^33^. This involved fitting two separate Cox models: one for CVD diagnoses and CVD deaths and one for non-CVD deaths with time from cohort entry as the underlying function^34^. We used fractional polynomials^35^ to model nonlinear risk relationships with continuous variables. We used multiple imputation with chained equations to replace missing values for ethnicity, BMI, SBP, total cholesterol, HDL and smoking status^36^. For binary variables, we coded them as present if there was a recorded diagnosis in the GP medical record and otherwise coded them as absent. We carried out five separate imputations for men and women in the derivation dataset. We included all predictor variables in the imputation model, along with age interaction terms, the Nelson–Aalen estimator of the CVD baseline cumulative hazard, the CVD outcome indicator, the baseline cumulative hazard and the outcome indicator for non-CVD death^29^. We combined results from Cox models using Rubin’s rules^37^. We included variables from existing QRISK3 models^8^ and retained additional variables with an adjusted hazard ratio of <0.90 or >1.10 (for binary variables) and statistical significance at the 0.01 level. We included significant interactions with age in the final model. We assessed model optimism by calculating heuristic shrinkage^16^. We combined estimates from the two cause-specific models to derive risk equations for the predicted risk of CVD at 10 years, accounting for competing events in men and women^34^.

We developed three additional models following peer review: Model A included the original QRISK3 parameters but did not account for competing risks; Model B was similar to our final model, but the follow-up time ended on 29 February 2020, before the COVID-19 pandemic; and Model C included time since cancer diagnosis as a predictor variable.

### Model evaluation

We also carried out multiple imputations, with five separate imputations for men and women in each validation cohort. We applied the risk equations to the internal and external validation cohorts and evaluated performance by country (England, Wales, Scotland and Northern Ireland).

We calculated concordance indices, equivalent to the C statistics, and accounted for competing risks^33^. We assessed model calibration, comparing the mean predicted risks at 10 years with the observed risks accounting for competing risks (cumulative incidence) by hundredths of predicted risk. We generated pseudo-values that accounted for competing risks in order to calculate the calibration slope and intercept at 10 years^38^.

We compared performance statistics for QR4 with those for ASCVD^3,39^, QRISK3 (ref. ^8^) and SCORE2 in England. We used the SCORE2 algorithm for those aged 40–69 years without diabetes^4^, SCORE2-OP for those 70 years and older without diabetes^7^ and SCORE2-Diabetes^40^ for those with diabetes using the authors’ published Stata code from July 2023 (ref. ^41^). We restricted comparisons between QR4, ASCVD and SCORE2 to people aged 40 years and older^3,4,6^. We also evaluated performance of QR4, ASCVD and SCORE2 using our secondary and tertiary outcomes.

### Decision curve analysis

We used decision curve analysis accounting for competing risks in both validation cohorts, in order to evaluate the net benefit of QR4 compared with that of QRISK3 and Model A and compared these with alternative strategies, such as assuming that all people were treated or nobody was treated^42^. The strategy with the highest net benefit at any given risk threshold was considered to have the most clinical value^43^. We also used decision curve analyses in people aged 40 years and older to compare QR4 with ASCVD and SCORE2 in the English validation cohort using all three outcomes.

### Reclassification statistics

We classified individuals as ‘high risk’ for CVD if their predicted 10-year risk was ≥10%, which is in line with current UK guidelines^5^. We compared the predicted risks of QR4 with those of QRISK3 and Model A to determine the percentage and characteristics of people reclassified at this high-risk threshold.

We also applied the predicted risk equations to men and women in the validation group to illustrate how each of the new risk factors affected 10-year CVD risk. In these calculations, men and women aged 18 to 84 were included, and CVD risk was compared between an individual with each of the new risk factors and a comparable reference individual but with no adverse clinical indicators (a cholesterol/HDL ratio of 4.0, an SBP of 125 mm Hg and a BMI of 25 kg m^−2^). For the example presented, we selected ‘White’ as the reference group, as this group had the largest number of participants.

We used all eligible individuals to develop and validate the models in order to maximize the power and generalizability of the results. We used Stata (version 17) for analyses.

### Inclusion and ethics

This study used anonymized data from two electronic health care records databases, and hence, participant consent was not required. The databases cover a diverse population that is representative of the UK population. The QResearch ethics approval was completed by the East Midlands-Derby Research Ethics Committee (reference 18/EM/0400). The CPRD ERAP approval reference is 20_000162.

### Reporting summary

Further information on research design is available in the Nature Portfolio Reporting Summary linked to this article.

## Online content

Any methods, additional references, Nature Portfolio reporting summaries, source data, extended data, supplementary information, acknowledgements, peer review information; details of author contributions and competing interests; and statements of data and code availability are available at 10.1038/s41591-024-02905-y.

### Supplementary information

Supplementary Information Supplementary Figs. 1–13 and Tables 1–4.

Reporting Summary

## Figures and Tables

**Extended Data Table 1:**
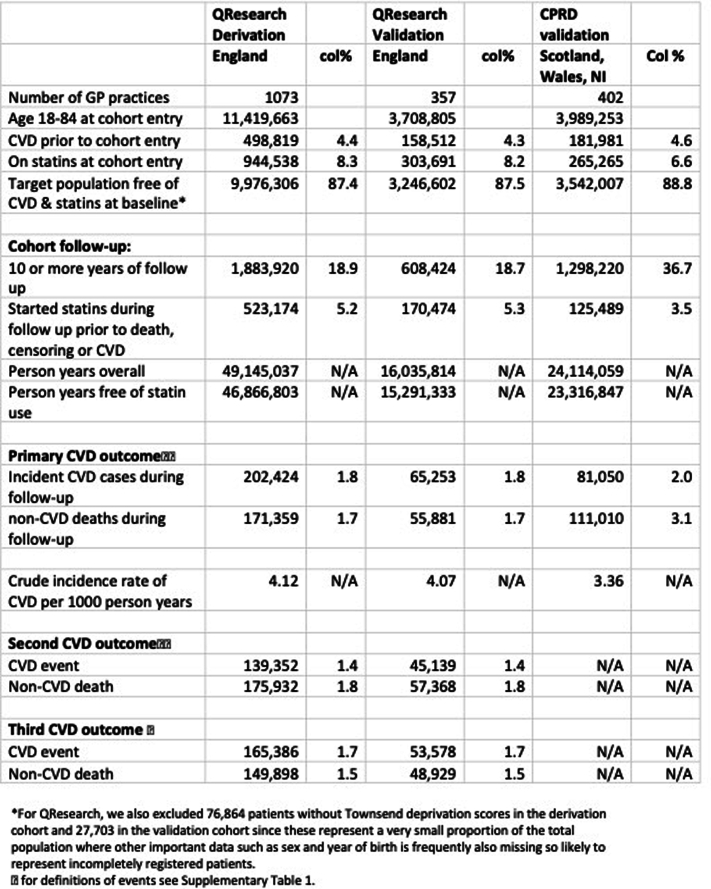
Flow of patients into the study for the QResearch Derivation Cohort and the validation cohorts from England and the remaining three nations (Wales, Scotland and Northern Ireland)

**Extended Data Table 2:**
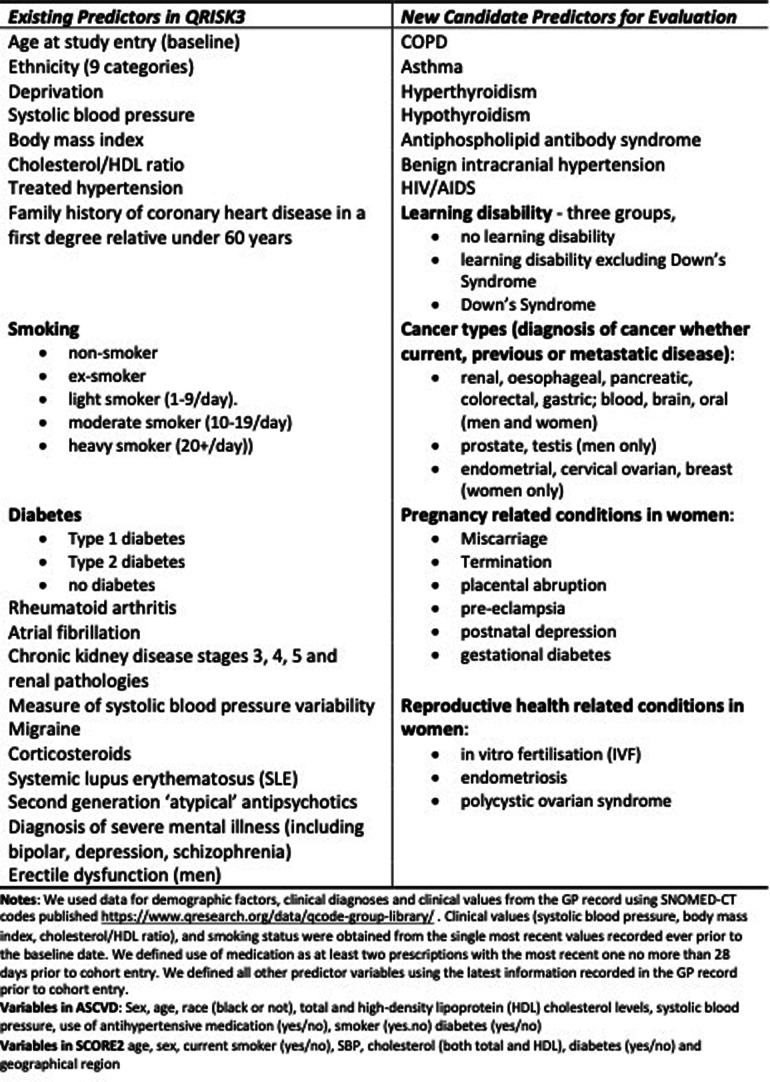
Existing predictors in QRISK3 and candidate predictors considered for inclusion in QR4

**Extended Data Table 3:**
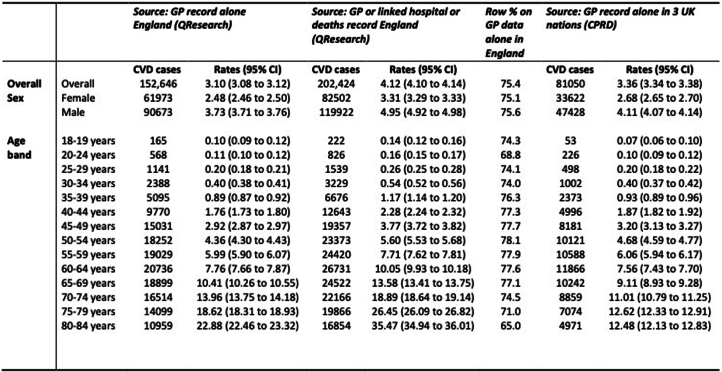
Number of CVD events and crude incidence rates per 1000 person years (95% CI) by age, sex and ethnicity in the QResearch derivation cohort in England compared with CPRD Gold validation cohort in other three UK nations (Scotland, Wales and Northern Ireland)

**Extended Data Table 4:**
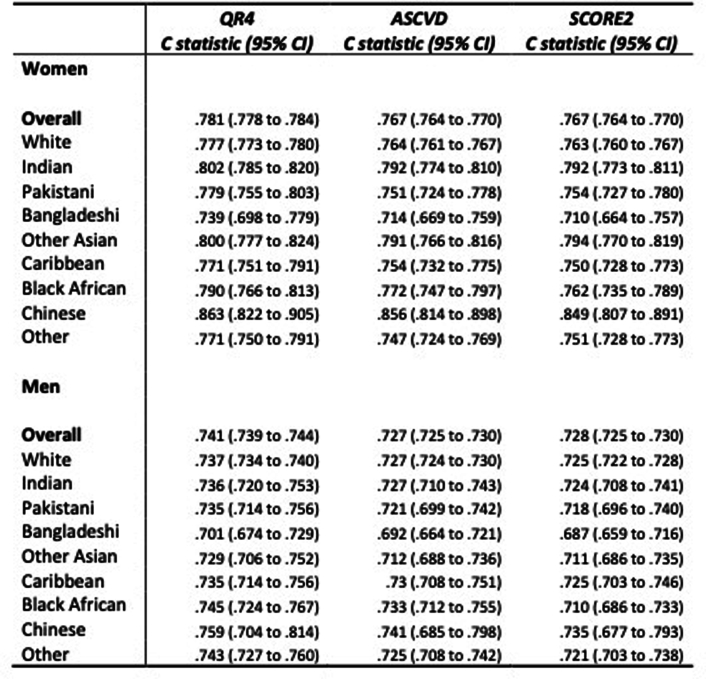
Discrimination using the C statistic (95% CI) for QR4, ASCVD and SCORE2 in people aged 40 and older in the England validation cohort overall and by ethnic group

**Extended Data Table 5:**
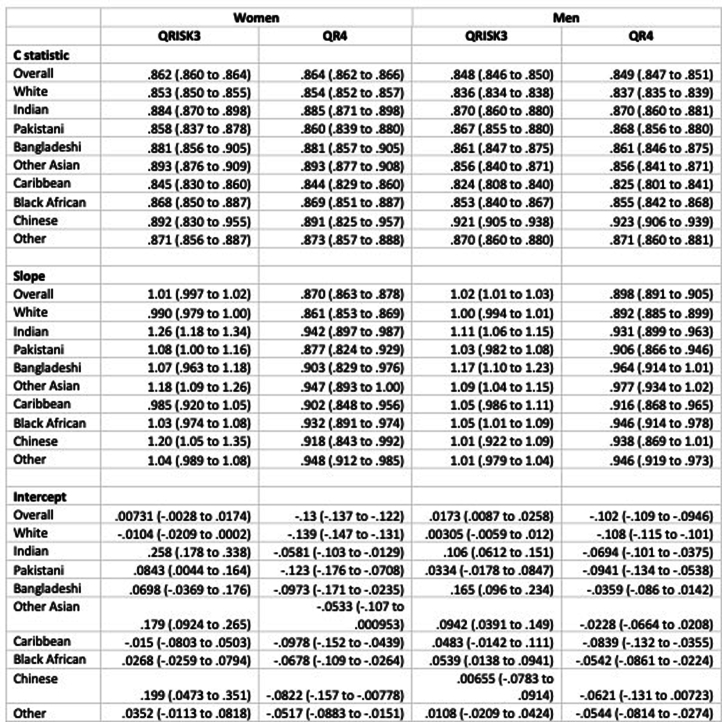
Discrimination using the C statistic (95% CI), calibration slope and intercept for QRISK3 and QR4 in the validation cohort in England by ethnic group in those aged 18–84 years

**Extended Data Table 6:**
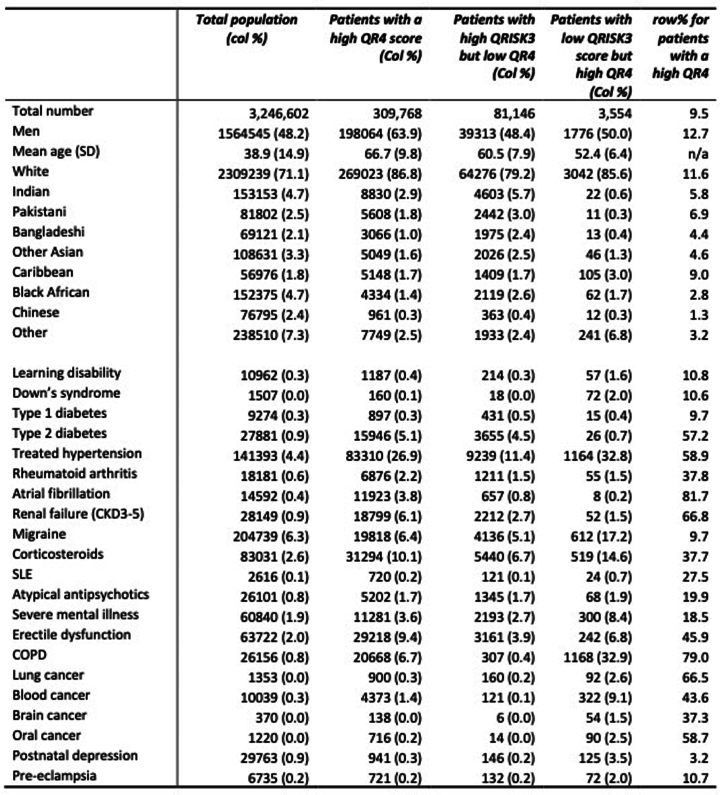
Characteristics of patients in the English validation cohort with a high-risk score (defined as 10-year risk of CVD of 10% or greater) including characteristics of those reclassified using QR4 compared with QRISK3

## Data Availability

To guarantee the confidentiality of personal and health information, only the authors had access to the data during the study in accordance with the relevant license agreements. The QResearch data are on the QResearch website (https://www.qresearch.org), and the CPRD data are on the CPRD website (https://www.cprd.com).

## Code availability

Clinical codes are published under a creative commons license at https://www.qresearch.org/data/qcode-group-library, with accompanying details in Supplementary Table 1. Software implementing the QR4 algorithm will be made available for research under an academic license by Oxford University Innovations (enquiries@innovation.ox.ac.uk). The QRISK3 algorithm is available at https://qrisk.org/src.php, and the ASCVD code is available at https://econpapers.repec.org/software/bocbocode/s459162.htm. SCORE2 algorithms are available at https://www.phpc.cam.ac.uk (ps.reception@medschl.cam.ac.uk).
