# Supplementary material for: Development and validation of a new algorithm for improved cardiovascular risk prediction
Source: Nat Med. 2024 Apr 18;30(5):1440–7. doi: 10.1038/s41591-024-02905-y (PMC11108771; doi:10.1038/s41591-024-02905-y)
Supplement: Supplementary file 1 — Supplementary Figs. 1–13 and Tables 1–4. [file 41591_2024_2905_MOESM1_ESM.pdf]

nature medicine

Article https://doi.org/10.1038/s41591-024-02905-y

# Development and validation of a new algorithm for improved cardiovascular risk prediction

In the format provided by the authors and unedited

# SUPPLEMENTARY FIGURES AND TABLES

## FIGURES

Supplementary Figure 1 Final model adjusted hazard ratios for non-CVD death in men and women, centered at the mean age of 39 for variables with age interactions and adjusted for FP terms for age, and BMI

Supplementary Figure 2 Adjusted hazard ratios for non-CVD death for Fractional polynomial terms for age and BMI and for age interactions.

Supplementary Figure 3 Adjusted hazard ratios for Model A (included original QRISK3 parameters without competing risks) for men and women.

Supplementary Figure 4 Adjusted hazard ratios for Model B (follow up time censored on 29.02.2020, immediately prior to the COVID-19 pandemic) for women.

Supplementary Figure 5 Adjusted hazard ratios for Model B (follow up time censored on 29.02.2020, immediately prior to the COVID-19 pandemic) for men.

Supplementary Figure 6 Adjusted hazard ratios for Model C (including time since diagnosis for cancer) for women.

Supplementary Figure 7 Adjusted hazard ratios for Model C (including time since diagnosis for cancer) for men.

Supplementary Figure 8 Predicted 10-year CVD risk using QR4 in patients with each of the new risk factors compared with an equivalent patient with the same values for other “less healthy” risk factors (systolic blood pressure=170 mm Hg; cholesterol ratio 6; BMI=35 kg/m2; light-smoker).

Supplementary Figure 9 Decision curves for QR4, ASCVD and SCORE2 in people aged 40+ in the England validation cohort using the second CVD outcome definition.

Supplementary Figure 10 Decision curves for QR4, ASCVD and SCORE2 in people aged 40+ in the England validation cohort using the third CVD outcome definition.

Supplementary Figure 11 Predicted and observed 10-year CVD risks for QR4 in people aged 18-84 years in Scotland, Wales and Northern Ireland using the primary CVD outcome definition.

Supplementary Figure 12 Predicted and observed 10-year CVD risks for QR4, ASCVD and SCORE2 in the validation cohort in England in people aged 40+, using the second CVD outcome definition.

Supplementary Figure 13 Predicted and observed 10-year CVD risks for QR4, ASCVD and SCORE2 in the validation cohort in England in people aged 40+ using the third CVD outcome definition.

# TABLES

Supplementary Table 1 CVD outcome definitions for sensitivity analyses.

Supplementary Table 2 Performance of QR4, SCORE2 and ASCVD in people age 40+ using the three CVD outcome measures in men and women in the validation cohort in England.

Supplementary Table 3 Characteristics of patients in the English validation cohort with a high QR4 risk score (defined as 10-year risk of CVD of 10% or greater) including characteristics of those reclassified using QR4 compared with Model A.

Supplementary Table 4: Characteristics of patients with complete vs missing data for patients aged 18-84 years in the English QResearch derivation cohort. Values are numbers (%) of patients unless indicated otherwise.

**Supplementary Figure 1 Final model adjusted hazard ratios for non-CVD death.** Shown are adjusted hazard ratios in 5,155,595 women and 4,820,711 men, presented at the mean age of 39 for variables with age interactions**.** The hazard ratios were adjusted for fractional polynomial terms for age, and BMI (see Supplementary Figure 1 which shows the relevant fractional polynomial terms. Systolic BP is per 20 unit increase.

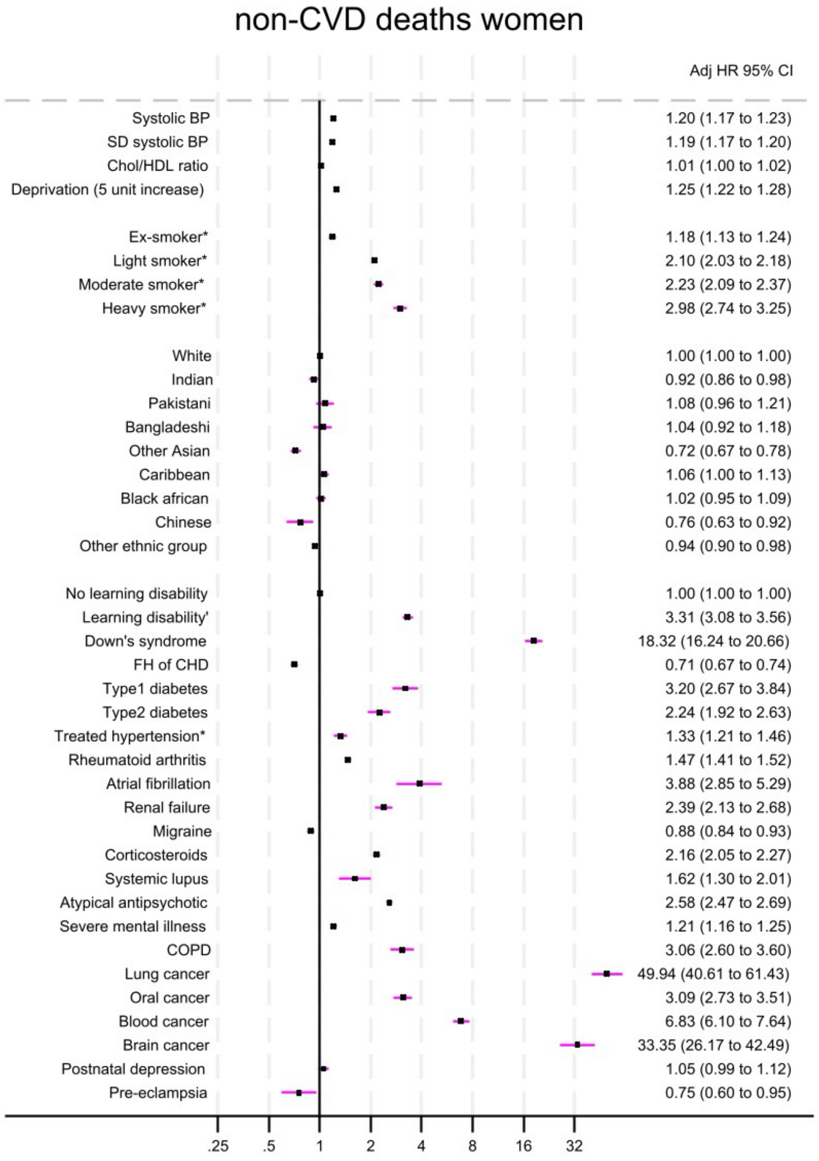


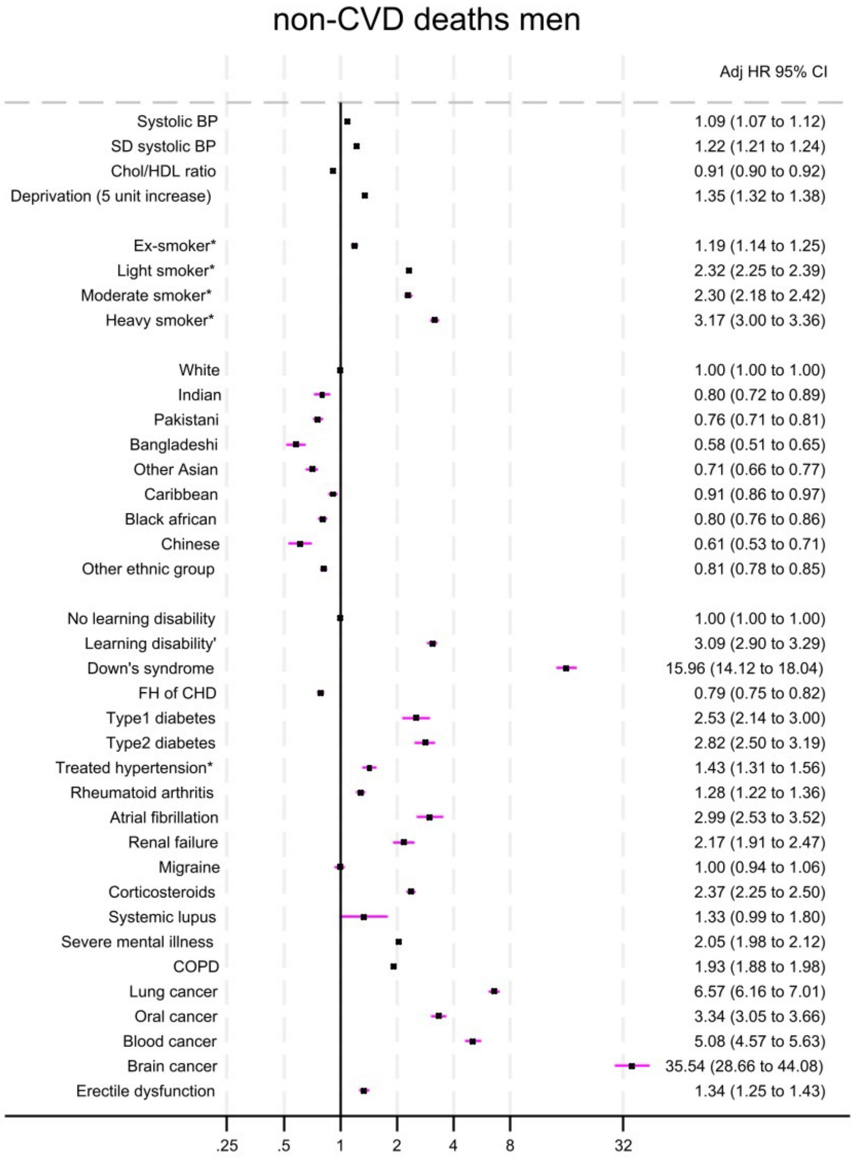

**Supplementary Figure 2 Adjusted hazard ratios for non-CVD death for fractional polynomial terms for and BMI, and for age interactions**

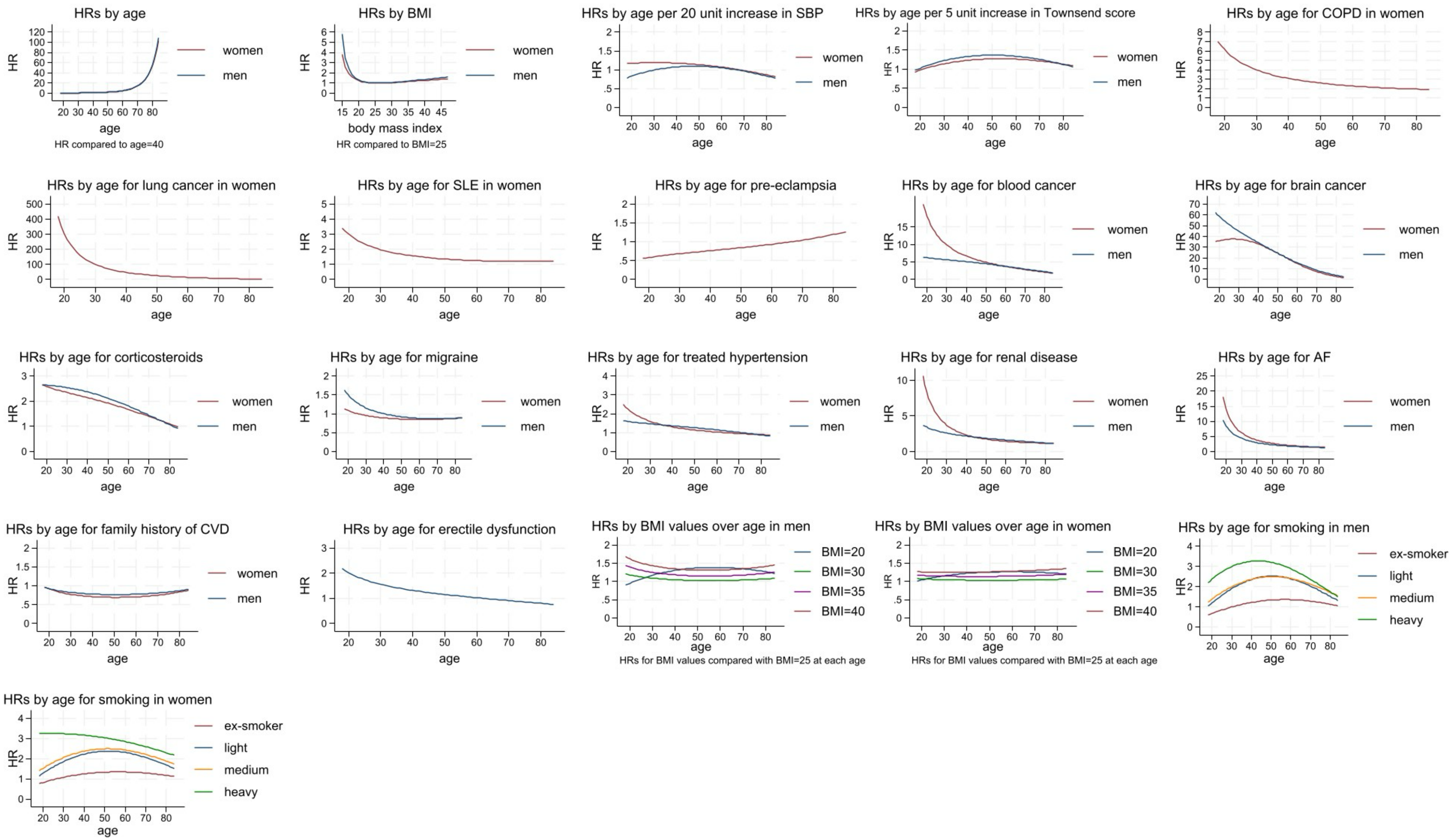

**Supplementary Figure 3 Adjusted mean hazard ratios (95% confidence intervals) for Model A in 4,820,711 men and 5,155,595 women.** Model A included original QRISK3 parameters without competing risks.

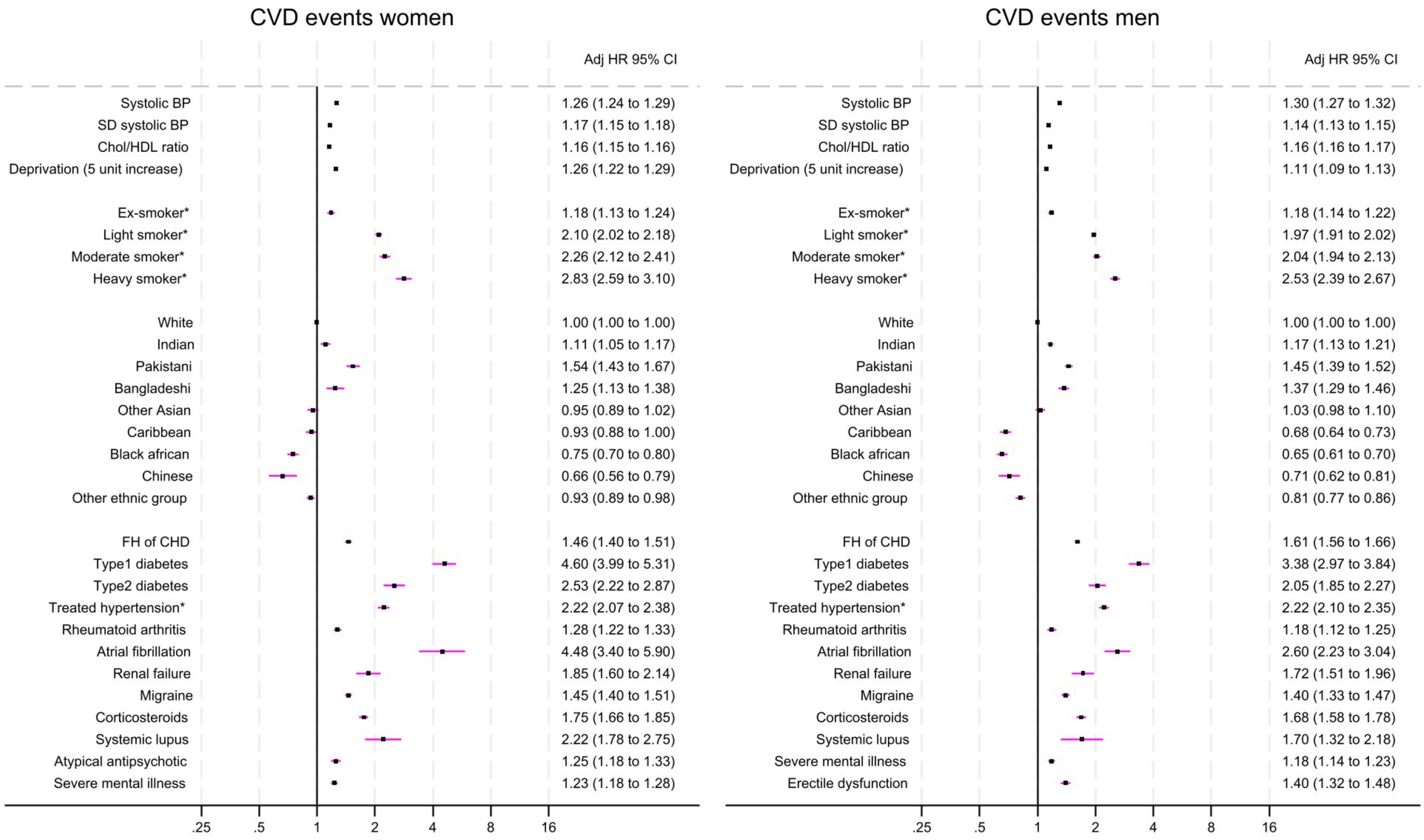

**Supplementary Figure 4 Adjusted mean hazard ratios (95% confidence intervals) for Model B in 5,155,595 women.** Model B had follow-up time censored on 29.02.2020, immediately prior to the COVID-19 pandemic.

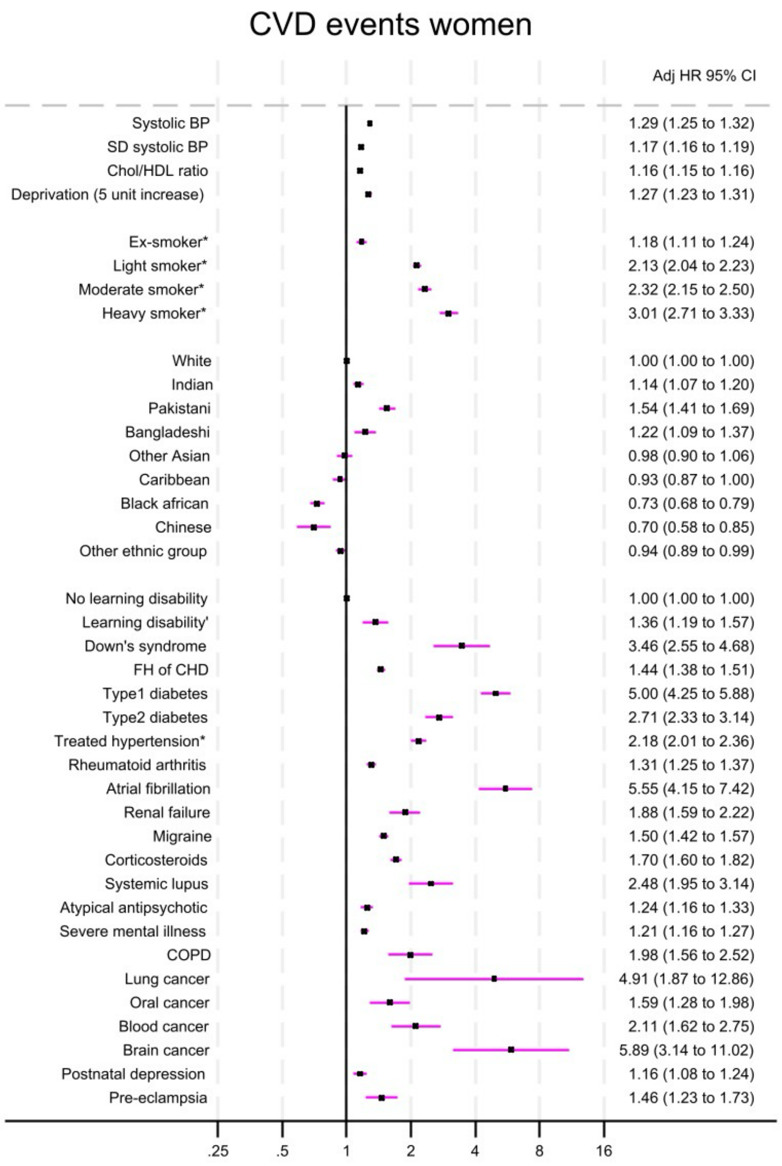


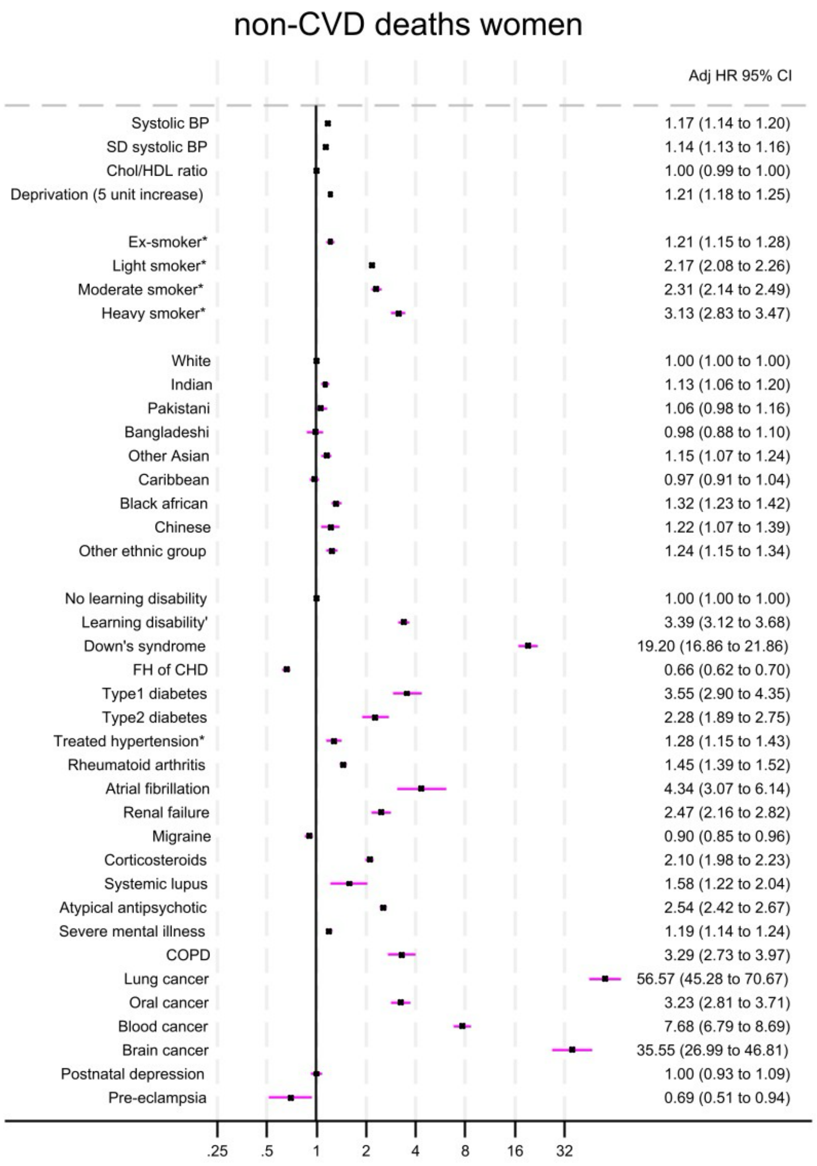

**Supplementary Figure 5 Adjusted mean hazard ratios (95% confidence intervals) for Model B in 4,820,711 men.** Model B had follow-up time censored on 29.02.2020, immediately prior to the COVID-19 pandemic.

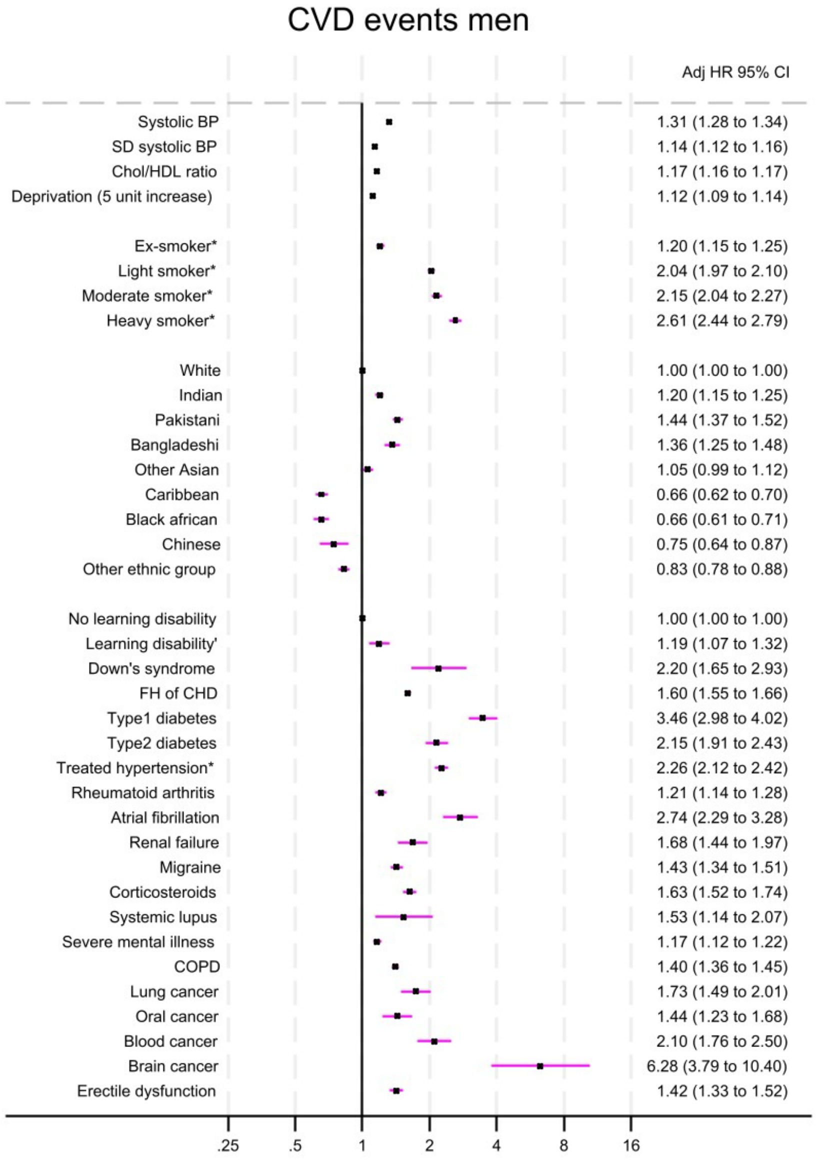


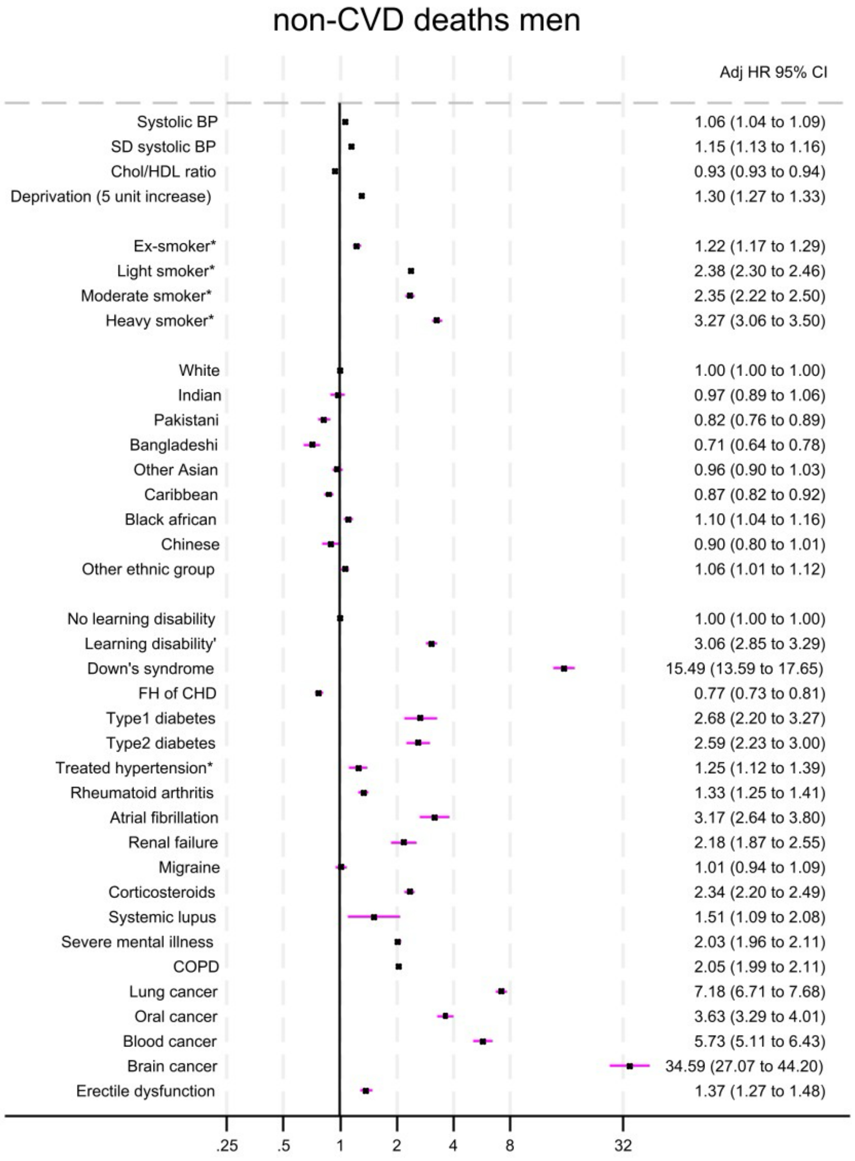

**Supplementary Figure 6 Adjusted mean hazard ratios (95% confidence intervals) for Model C in 5,155,595 women.** Model C includes time since diagnosis for cancer.

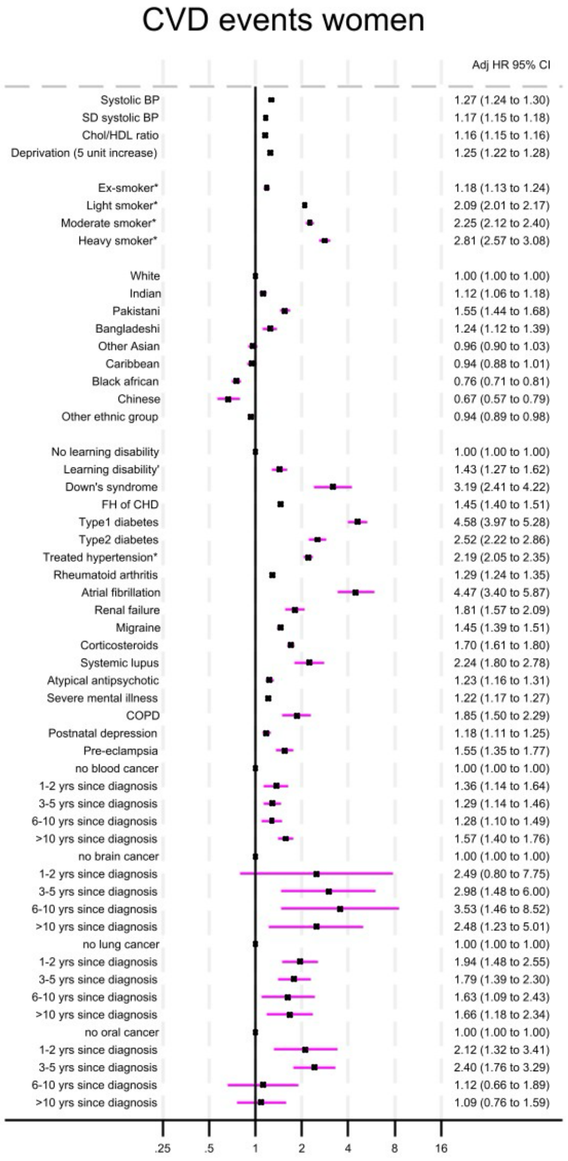


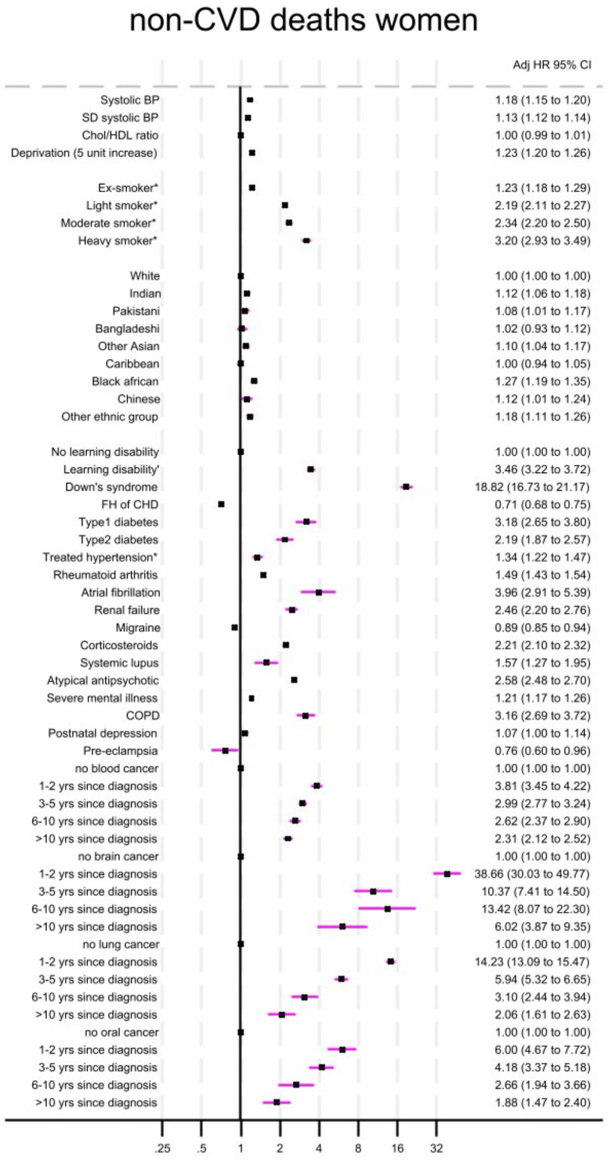

**Supplementary Figure 7 Adjusted mean hazard ratios (95% confidence intervals) for Model C.** Model C includes time since diagnosis for cancer for 4,820,711 men.

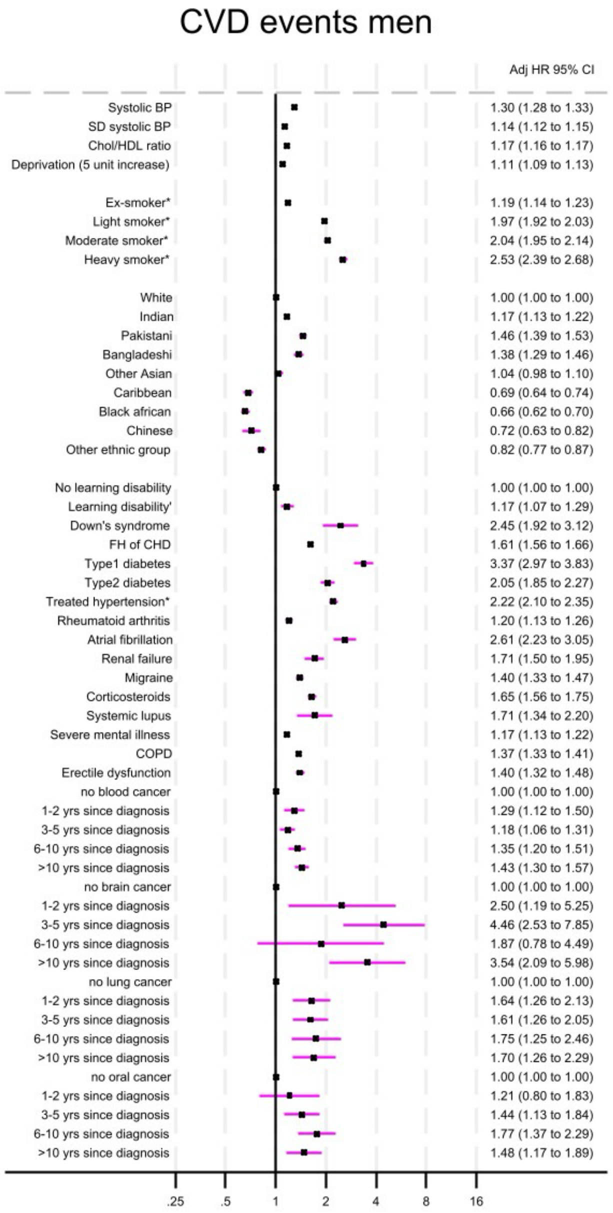


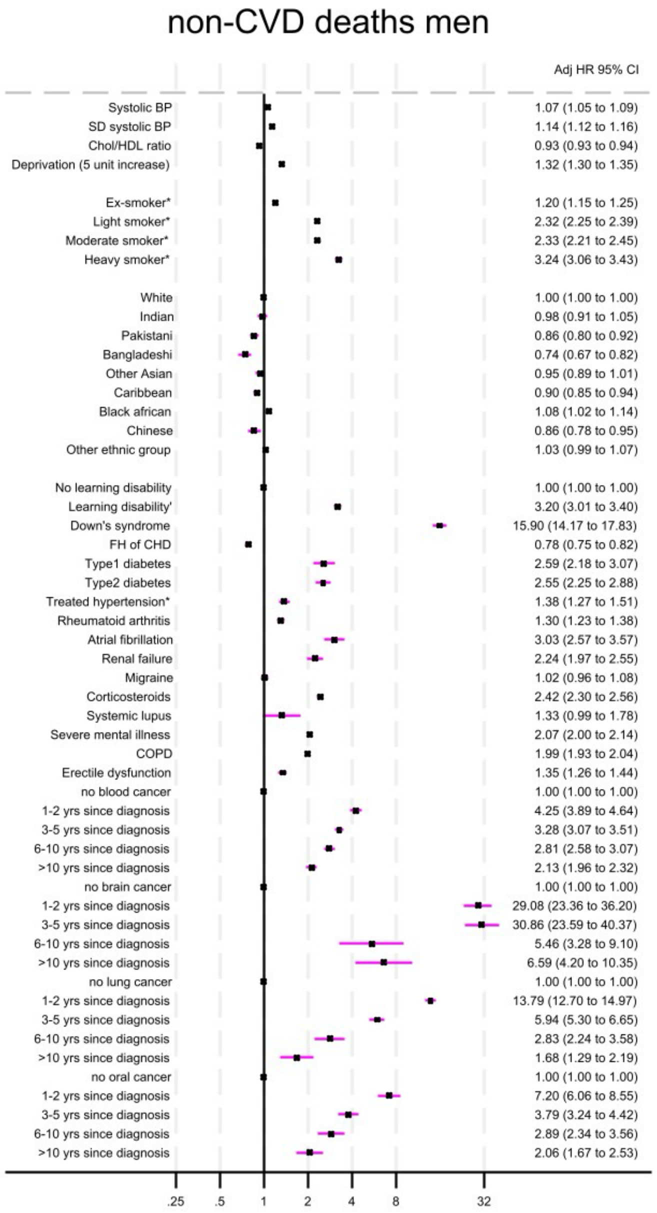

**Supplementary Figure 8 Predicted 10-year CVD risk using QR4 in patients with each of the new risk factors compared with a equivalent patient with "less healthy" risk factors (systolic blood pressure=170 mm Hg; cholesterol ratio 6; BMI=35 kg/m2; light-smoker) but no adverse clinical conditions.**

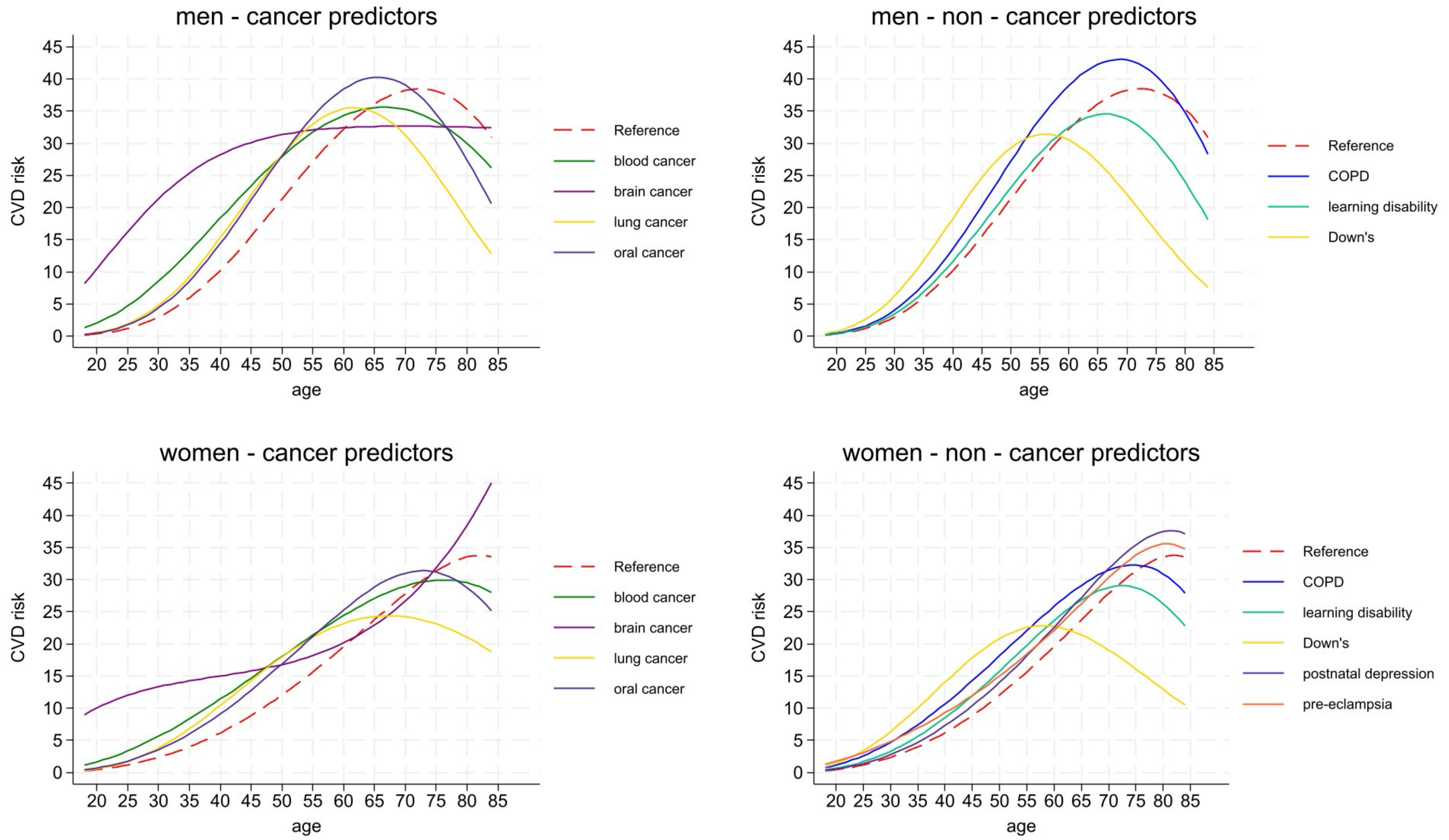

**Supplementary Figure 9 Decision curves for QR4, ASCVD and SCORE2 in people aged 40+ in the England validation cohort using the second CVD outcome definition.**

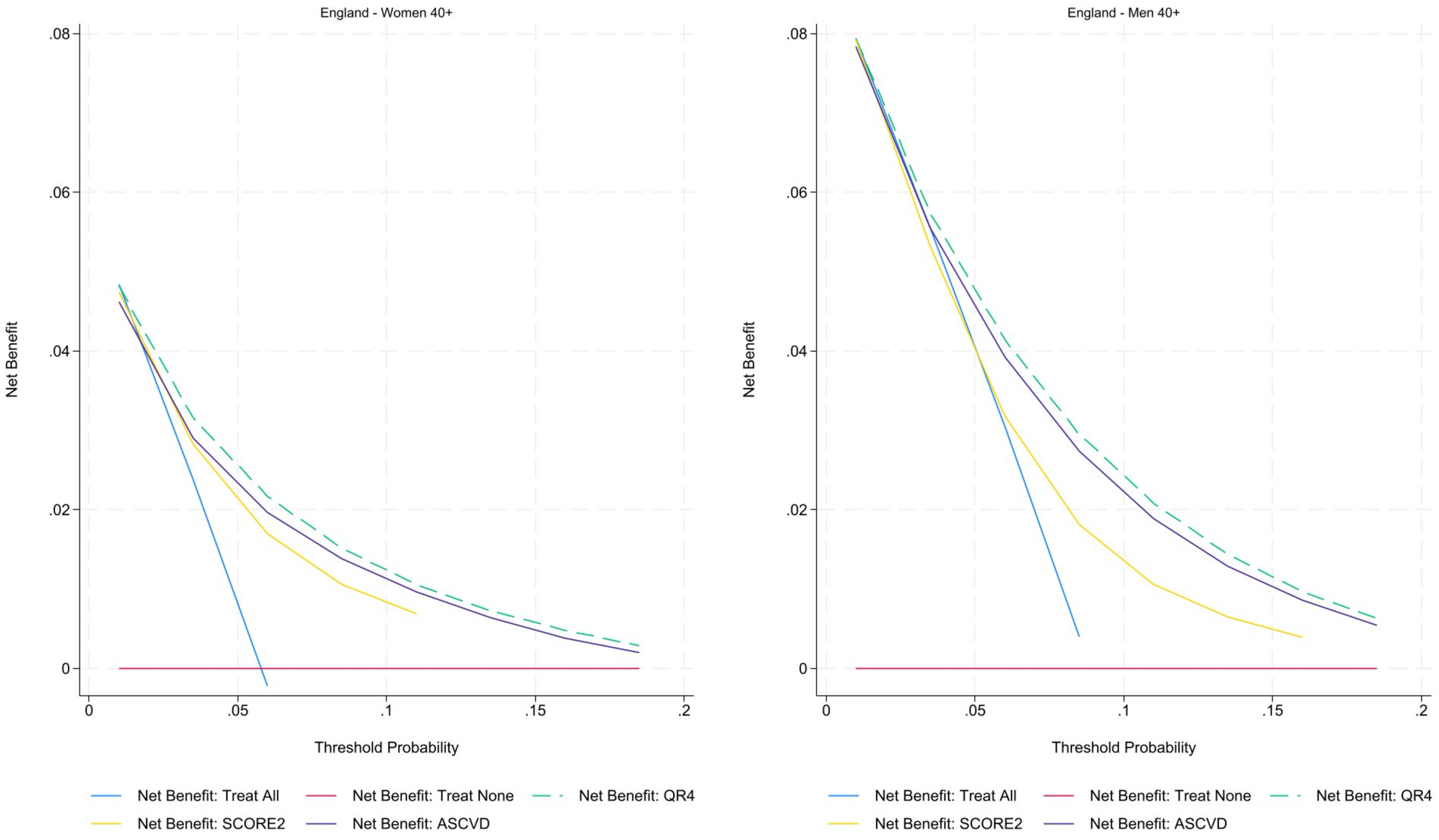

**Supplementary Figure 10 Decision curves for QR4, ASCVD and SCORE2 in people aged 40+ in the England validation cohort using the third CVD outcome.**

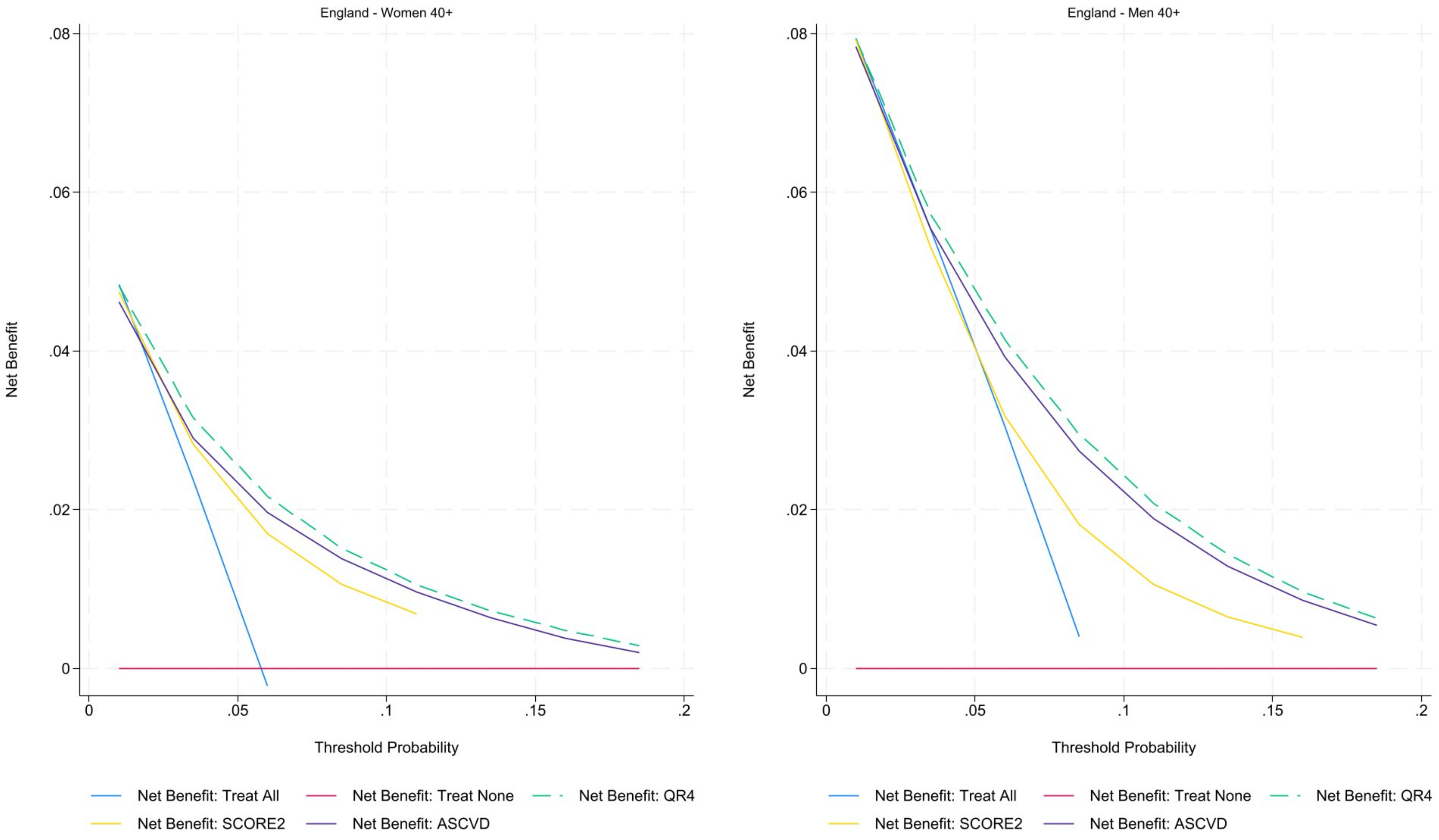

**Supplementary Figure 11 Predicted and observed 10-year CVD risks for QR4, in people aged 18-84 in Scotland, Wales and Northern Ireland validation cohorts using the primary CVD outcome definition.**

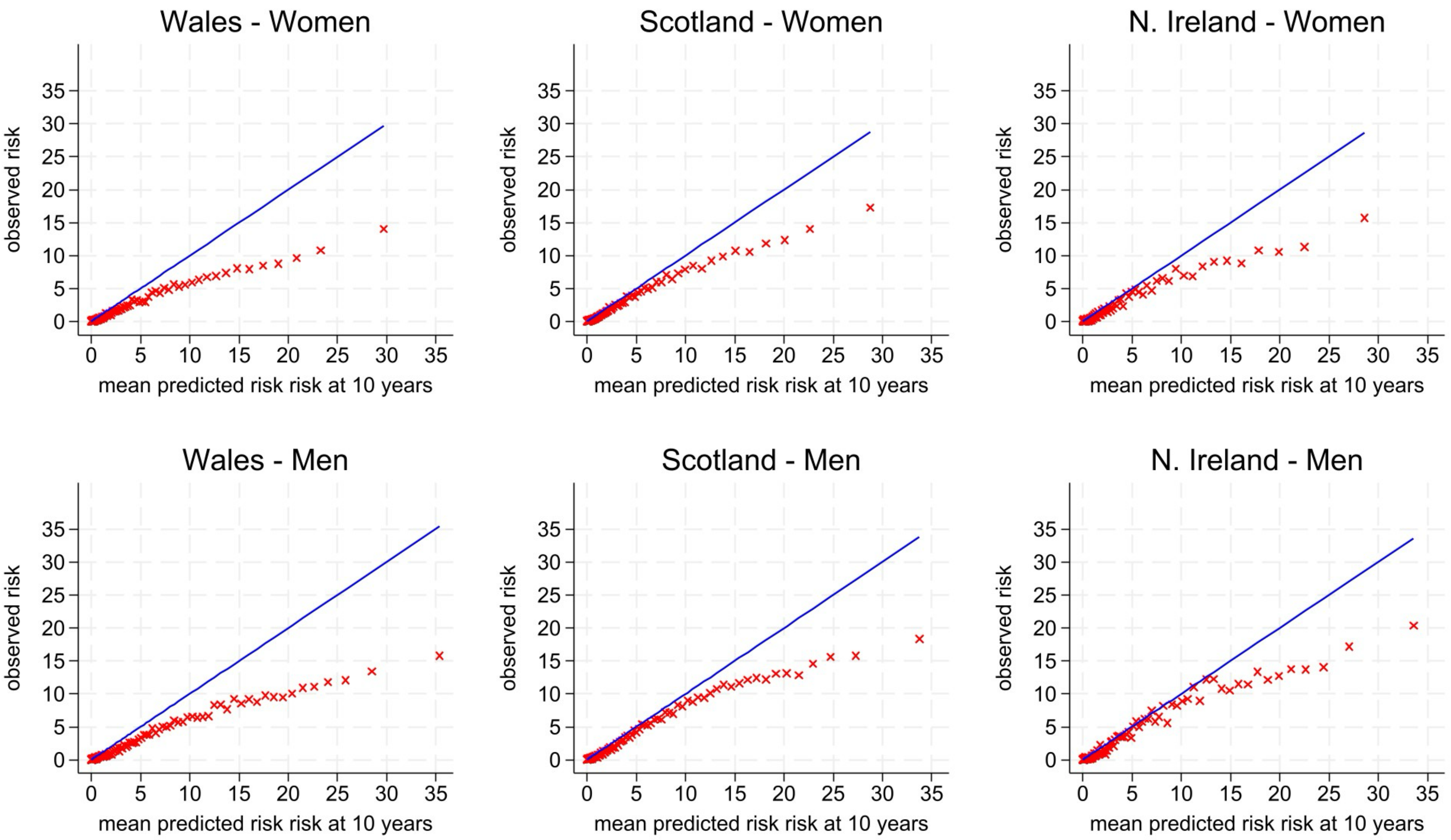

**Supplementary Figure 12 Predicted and observed 10-year CVD risks for QR4, ASCVD and SCORE2 in the England validation cohort for people aged 40+ using the second CVD outcome definition.**

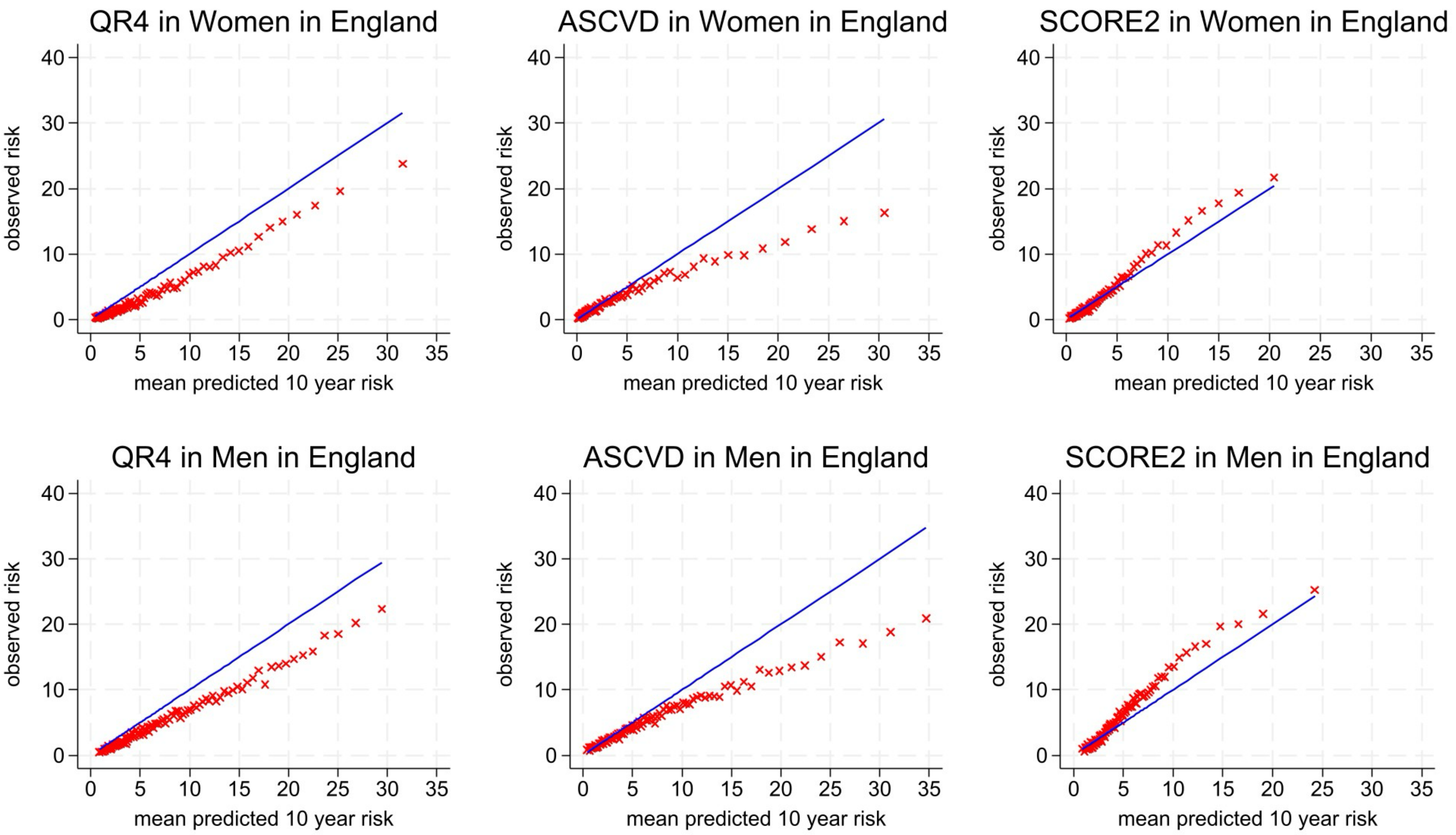

**Supplementary Figure 13 Predicted and observed 10-year CVD risks for QR4, ASCVD and SCORE2 in the validation cohort in England, using the third CVD outcome definition**

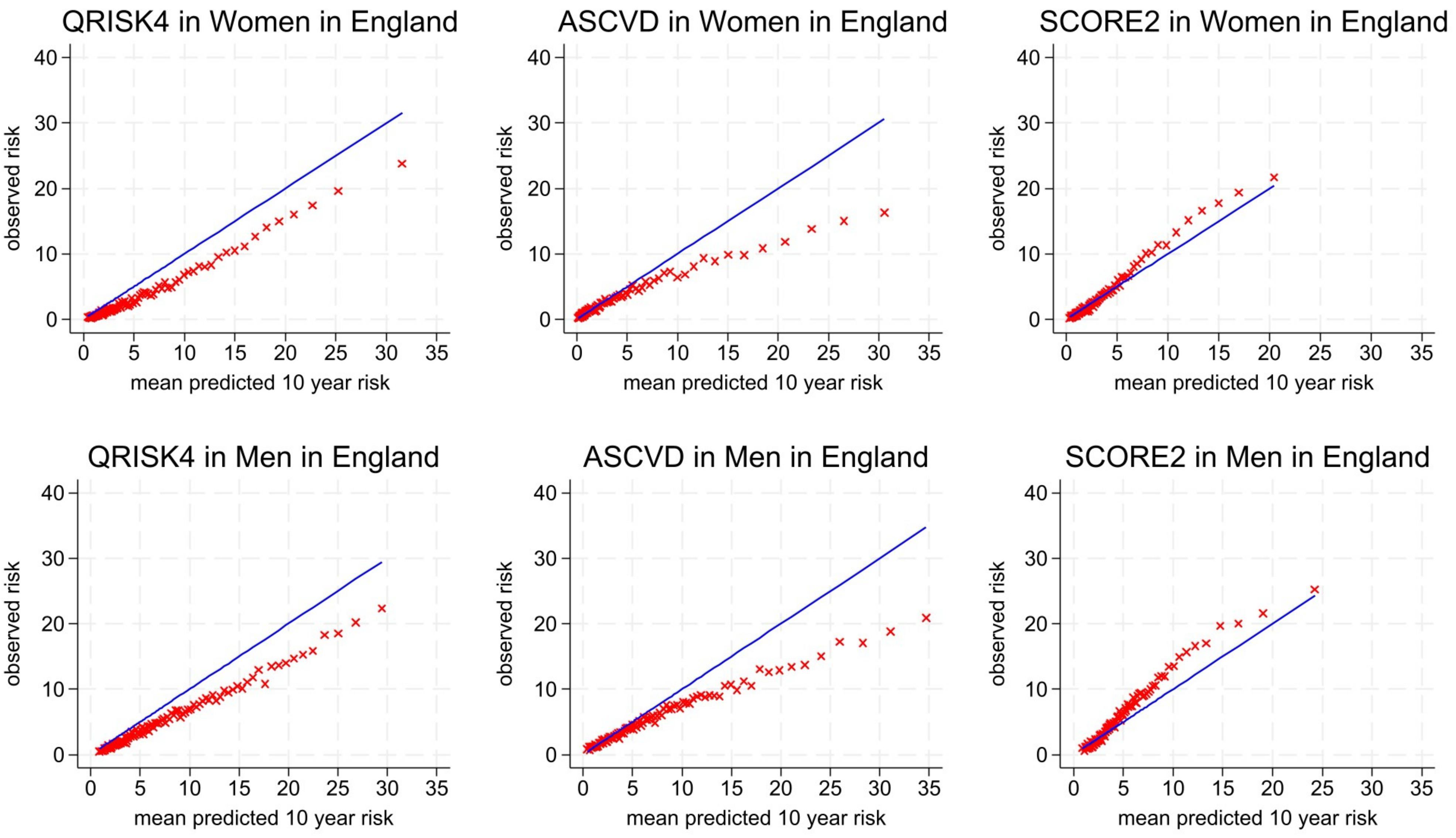

**Supplementary Table 1 CVD outcome definitions for QR4 model development and the definitions aligned to SCORE2 and ASCVD.**

| | | | |
|---|---|---|---|
| *QR4* | ***Fatal or non-fatal cardiovascular disease*** | | *ICD10-codes* |
| | *End points included* | Ischemic heart disease | I20-25 |
| | | Cerebrovascular disease | I60-69 |
| | | Transient ischaemic attack | G45 |
| | | | |
| | Endpoints excluded | Subarachnoid haemorrhage | I60 |
| | | Subdural haemorrhage | I62 |
| | | Cerebral aneurysm | I67.1 |
| | | Cerebral arteritis | I68.2 |
| | | Moyamoya | I67.5 |
| | | | |
| *ASCVD* | ***Fatal cardiovascular disease*** | | |
| | *Endpoints included* | Ischemic heart disease | I20-25 |
| | | Cerebrovascular disease | I60-69 |
| | | | |
| | *Endpoints excluded* | Subarachnoid haemorrhage | I60 |
| | | Subdural haemorrhage | I62 |
| | | Cerebral aneurysm | I67.1 |
| | | Cerebral arteritis | I68.2 |
| | | Moyamoya | I67.5 |
| | | | |
| | ***Non-fatal cardiovascular disease*** | | |
| | *Endpoints included* | Non-fatal myocardial infarction | I21-I23 |
| | | Non-fatal stroke | I60-69 |
| | *Endpoints excluded* | Subarachnoid haemorrhage | I60 |
| | | Subdural haemorrhage | I62 |
| | | Cerebral aneurysm | I67.1 |
| | | Cerebral arteritis | I68.2 |
| | | Moyamoya | I67.5 |
| | | | |
| *SCORE2* | ***Fatal cardiovascular disease*** | | |
| | *Endpoints included* | Hypertensive disease | I10-16 |
| | | Ischemic heart disease | I20-25 |
| | | Arrhythmias, heart failure | I46-52 |
| | | Cerebrovascular disease | I60-69 |
| | | Atherosclerosis/AAA | I70-73 |
| | | Sudden death and death within 24h of symptom onset | R96.0-96.1 |
| | *Endpoints excluded* | Myocarditis, unspecified | I51.4 |
| | | Subarachnoid haemorrhage | I60 |
| | | Subdural haemorrhage | I62 |
| | | Cerebral aneurysm | I67.1 |
| | | Cerebral arteritis | I68.2 |
| | | Moyamoya | I67.5 |
| | | | |
| | ***Non-fatal cardiovascular disease*** | | |
| | *Endpoints included* | Non-fatal myocardial infarction | I21-I23 |
| | | Non-fatal stroke | I60-69 |
| | | | |
| | *Endpoints excluded* | Subarachnoid haemorrhage | I60 |
| | | Subdural haemorrhage | I62 |
| | | Cerebral aneurysm | I67.1 |
| | | Cerebral arteritis | I68.2 |
| | | Moyamoya | I67.5 |

**Supplementary Table 2 Performance of QR4, SCORE2 and ASCVD in 3,246,602 people age 40+ using three different CVD outcome definitions in the England validation cohort.**

| | Women QR4 mean (95% CI) | Women ASCVD mean (95% CI) | Women SCORE2 mean (95% CI) | Men QR4 mean (95% CI) | Men ASCVD mean (95% CI) | Men SCORE2 mean (95% CI) |
|---|---|---|---|---|---|---|
| | | | | | | |
| | | | | | | |
| **Outcome 1** | | | | | | |
| C statistic | .781 (.778 to .784) | .767 (.764 to .770) | .767 (.764 to .770) | .741 (.739 to .744) | .727 (.725 to .730) | .728 (.725 to .730) |
| Slope | .870 (.863 to .878) | .869 (.860 to .878) | .733 (.727 to .739) | .898 (.891 to .905) | .900 (.893 to .907) | .720 (.715 to .725) |
| Intercept | -.130 (-.137 to -.122) | -.131 (-.140 to -.122) | -.267 (-.273 to -.261) | -.102 (-.109 to -.0946) | -.100 (-.107 to -.0926) | -.280 (-.285 to -.275) |
| | | | | | | |
| **Outcome 2** | | | | | | |
| C statistic | .790 (.787 to .794) | .777 (.773 to .781) | .778 (.774 to .781) | .742 (.739 to .745) | .729 (.726 to .732) | .730 (.727 to .733) |
| Slope | 1.16 (1.15 to 1.17) | 1.39 (1.37 to 1.40) | .936 (.929 to .943) | 1.16 (1.16 to 1.17) | 1.22 (1.21 to 1.23) | .891 (.885 to .896) |
| Intercept | .162 (.152 to .172) | .386 (.368 to .403) | -.0642 (-.0712 to -.0573) | .164 (.156 to .173) | .223 (.213 to .233) | -.109 (-.115 to -.104) |
| | | | | | | |
| **Outcome 3** | | | | | | |
| C statistic | .793 (.790 to .797) | .780 (.776 to .783) | .780 (.777 to .784) | .744 (.741 to .747) | .731 (.728 to .735) | .732 (.729 to .735) |
| Slope | 1.03 (1.02 to 1.04) | 1.12 (1.11 to 1.13) | .846 (.840 to .851) | 1.07 (1.06 to 1.08) | 1.11 (1.10 to 1.11) | .831 (.826 to .836) |
| Intercept | .0304 (.0224 to .0384) | .117 (.105 to .128) | -.154 (-.160 to -.149) | .0692 (.0618 to .0767) | .106 (.0974 to .114) | -.169 (-.174 to -.164) |
| | | | | | | |

**Supplementary Table 3. Characteristics of 3,246,602 patients in the English validation cohort with a high QR4 risk score (defined as 10-year risk of CVD of 10% or greater) including characteristics of those reclassified using QR4 compared with Model A.**

| | *Total population (col %)* | *Patients with a high QR4 score (Col %)* | *Patients with high Model A but low QR4 (Col %)* | *Patients with low Model A score but high QR4 (Col %)* | *row% for patients with a high QR4* |
|---|---|---|---|---|---|
| Total number | 3,246,602 | 309,768 | 12791 | 4068 | 9.5 |
| Men | 1564545 (48.2) | 198064 (63.9) | 5961 (46.6) | 1790 (44.0) | 12.7 |
| Mean age (SD) | 38.9 (14.9) | 66.7 (9.8) | 61.2 (8.1) | 56.8 (7.9) | n/a |
| White | 2309239 (71.1) | 269023 (86.8) | 10951 (85.6) | 3529 (86.8) | 11.6 |
| Indian | 153153 (4.7) | 8830 (2.9) | 147 (1.1) | 105 (2.6) | 5.8 |
| Pakistani | 81802 (2.5) | 5608 (1.8) | 117 (0.9) | 68 (1.7) | 6.9 |
| Bangladeshi | 69121 (2.1) | 3066 (1.0) | 41 (0.3) | 75 (1.8) | 4.4 |
| Other Asian | 108631 (3.3) | 5049 (1.6) | 147 (1.1) | 72 (1.8) | 4.6 |
| Caribbean | 56976 (1.8) | 5148 (1.7) | 453 (3.5) | 58 (1.4) | 9.0 |
| Black African | 152375 (4.7) | 4334 (1.4) | 399 (3.1) | 48 (1.2) | 2.8 |
| Chinese | 76795 (2.4) | 961 (0.3) | 61 (0.5) | 8 (0.2) | 1.3 |
| Other | 238510 (7.3) | 7749 (2.5) | 475 (3.7) | 105 (2.6) | 3.2 |
| | | | | | |
| Learning disability | 10962 (0.3) | 1187 (0.4) | 46 (0.4) | 180 (4.4) | 10.8 |
| Down’s syndrome | 1507 (0.0) | 160 (0.1) | 12 (0.1) | 119 (2.9) | 10.6 |
| Type 1 diabetes | 9274 (0.3) | 897 (0.3) | 44 (0.3) | 20 (0.5) | 9.7 |
| Type 2 diabetes | 27881 (0.9) | 15946 (5.1) | 475 (3.7) | 91 (2.2) | 57.2 |
| Treated hypertension | 141393 (4.4) | 83310 (26.9) | 2084 (16.3) | 567 (13.9) | 58.9 |
| Rheumatoid arthritis | 18181 (0.6) | 6876 (2.2) | 269 (2.1) | 64 (1.6) | 37.8 |
| Atrial fibrillation | 14592 (0.4) | 11923 (3.8) | 72 (0.6) | 23 (0.6) | 81.7 |
| Renal failure (CKD3-5) | 28149 (0.9) | 18799 (6.1) | 408 (3.2) | 110 (2.7) | 66.8 |
| Migraine | 204739 (6.3) | 19818 (6.4) | 672 (5.3) | 435 (10.7) | 9.7 |
| Corticosteroids | 83031 (2.6) | 31294 (10.1) | 2046 (16.0) | 888 (21.8) | 37.7 |
| SLE | 2616 (0.1) | 720 (0.2) | 21 (0.2) | 10 (0.2) | 27.5 |
| Atypical antipsychotics | 26101 (0.8) | 5202 (1.7) | 403 (3.2) | 111 (2.7) | 19.9 |
| Severe mental illness | 60840 (1.9) | 11281 (3.6) | 732 (5.7) | 241 (5.9) | 18.5 |
| Erectile dysfunction | 63722 (2.0) | 29218 (9.4) | 468 (3.7) | 178 (4.4) | 45.9 |
| COPD | 26156 (0.8) | 20668 (6.7) | 143 (1.1) | 2120 (52.1) | 79.0 |
| Lung cancer | 1353 (0.0) | 900 (0.3) | 151 (1.2) | 153 (3.8) | 66.5 |
| Blood cancer | 10039 (0.3) | 4373 (1.4) | 34 (0.3) | 712 (17.5) | 43.6 |
| Brain cancer | 370 (0.0) | 138 (0.0) | 6 (0.0) | 67 (1.6) | 37.3 |
| Oral cancer | 1220 (0.0) | 716 (0.2) | 12 (0.1) | 157 (3.9) | 58.7 |
| Postnatal depression | 29763 (0.9) | 941 (0.3) | 0 (0.0) | 225 (5.5) | 3.2 |
| Pre-eclampsia | 6735 (0.2) | 721 (0.2) | 0 (0.0) | 136 (3.3) | 10.7 |

**Supplementary Table 4: Characteristics of patients with complete vs missing data for patients aged 18-84 years in the English QResearch derivation cohort**. Values are numbers (%) of patients unless indicated otherwise. Complete data is defined as values recorded for all of ethnicity, cholesterol/HDL ratio, BMI, smoking, systolic blood pressure. Diagnoses are assumed to be not present if not recorded.

| | *not complete data* | *complete data* |
|---|---|---|
| total patients | 8223690 | 1752616 |
| men | 4035820 (49.1) | 784891 (44.8) |
| mean age(SD) | 36.8 (14.3) | 49.4 (14.0) |
| mean Townsend score (SD) | 0.8 (3.2) | 0.5 (3.2) |
| BMI recorded | 5997020 (72.9) | 1752616 (100.0) |
| Chol/HDL ratio recorded | 1075677 (13.1) | 1752616 (100.0) |
| SBP recorded | 6247094 (76.0) | 1752616 (100.0) |
| mean BMI (SD) | 25.1 (5.0) | 27.3 (5.5) |
| mean chol/HDL (SD) | 3.8 (1.2) | 3.8 (1.2) |
| mean SBP (SD) | 122.6 (15.2) | 127.1 (15.5) |
| | | |
| Ethnicity recorded | 4433551 (53.9) | 1752616 (100.0) |
| White | 3106755 (37.8) | 1284387 (73.3) |
| Indian | 213781 (2.6) | 87633 (5.0) |
| Pakistani | 127703 (1.6) | 58326 (3.3) |
| Bangladeshi | 84312 (1.0) | 31370 (1.8) |
| Other Asian | 162334 (2.0) | 56221 (3.2) |
| Caribbean | 66717 (0.8) | 36861 (2.1) |
| Black African | 199902 (2.4) | 85424 (4.9) |
| Chinese | 133855 (1.6) | 14924 (0.9) |
| Other | 338192 (4.1) | 97470 (5.6) |
| | | |
| Smoking status recorded | 7673710 (93.3) | 1752616 (100.0) |
| non smoker | 4700431 (57.2) | 1063711 (60.7) |
| Ex-smoker | 1218807 (14.8) | 381554 (21.8) |
| light smoker | 1355030 (16.5) | 234086 (13.4) |
| moderate smoker | 279321 (3.4) | 47897 (2.7) |
| heavy smoker | 120121 (1.5) | 25368 (1.4) |
| | | |
| Learning disability | 25487 (0.3) | 9176 (0.5) |
| Down's syndrome | 3599 (0.0) | 1218 (0.1) |
| Type 1 diabetes | 13236 (0.2) | 15043 (0.9) |
| Type 2 diabetes | 32771 (0.4) | 54993 (3.1) |
| Treated hypertension | 201191 (2.4) | 232912 (13.3) |
| Rheumatoid arthritis | 36767 (0.4) | 20991 (1.2) |
| Atrial fibrillation | 28100 (0.3) | 18085 (1.0) |
| Renal failure (CKD3-5) | 49425 (0.6) | 38727 (2.2) |
| Migraine | 484195 (5.9) | 149991 (8.6) |
| Corticosteroids | 173306 (2.1) | 82901 (4.7) |
| SLE | 5180 (0.1) | 2687 (0.2) |
| Atypical antipsychotics | 51286 (0.6) | 28600 (1.6) |
| Severe mental illness | 133819 (1.6) | 62937 (3.6) |
| Erectile dysfunction | 117648 (1.4) | 80655 (4.6) |
| COPD | 47863 (0.6) | 32128 (1.8) |
| Lung cancer | 2941 (0.0) | 1481 (0.1) |

| | | |
|---|---|---|
| Blood cancer | 21890 (0.3) | 9119 (0.5) |
| Brain cancer | 958 (0.0) | 287 (0.0) |
| Oral cancer | 2564 (0.0) | 1300 (0.1) |
| Postnatal depression | 76100 (0.9) | 20363 (1.2) |
| Pre-eclampsia | 14479 (0.2) | 5754 (0.3) |
