# Supplementary material for: Development and validation of a new algorithm for improved cardiovascular risk prediction
Source: Nat Med. 2024 Apr 18;30(5):1440–7. doi: 10.1038/s41591-024-02905-y (PMC11108771; doi:10.1038/s41591-024-02905-y)
Supplement: Supplementary file 2 — Reporting Summary [file 41591_2024_2905_MOESM2_ESM.pdf]

nature portfolio

Corresponding author(s): Julia Hippisley-Cox

Last updated by author(s): 28/02/2024

Reporting Summary

Nature Portfolio wishes to improve the reproducibility of the work that we publish. This form provides structure for consistency and transparency in reporting. For further information on Nature Portfolio policies, see our Editorial Policies and the Editorial Policy Checklist.

Statistics

For all statistical analyses, confirm that the following items are present in the figure legend, table legend, main text, or Methods section.

n/a	Confirmed	
☐	☒	The exact sample size (n) for each experimental group/condition, given as a discrete number and unit of measurement
☐	☒	A statement on whether measurements were taken from distinct samples or whether the same sample was measured repeatedly
☐	☒	The statistical test(s) used AND whether they are one- or two-sided<br>*Only common tests should be described solely by name; describe more complex techniques in the Methods section.*
☐	☒	A description of all covariates tested
☐	☒	A description of any assumptions or corrections, such as tests of normality and adjustment for multiple comparisons
☐	☒	A full description of the statistical parameters including central tendency (e.g. means) or other basic estimates (e.g. regression coefficient) AND variation (e.g. standard deviation) or associated estimates of uncertainty (e.g. confidence intervals)
☒	☐	For null hypothesis testing, the test statistic (e.g. F, t, r) with confidence intervals, effect sizes, degrees of freedom and P value noted<br>*Give P values as exact values whenever suitable.*
☒	☐	For Bayesian analysis, information on the choice of priors and Markov chain Monte Carlo settings
☐	☒	For hierarchical and complex designs, identification of the appropriate level for tests and full reporting of outcomes
☒	☐	Estimates of effect sizes (e.g. Cohen's d, Pearson's r), indicating how they were calculated

Our web collection on statistics for biologists contains articles on many of the points above.

Software and code

Policy information about availability of computer code

Data collection	no software was used for data collection
Data analysis	Stata version 18. QRISK3 source code is available at https://qrisk.org/src.php. ASCVD code is available from https://econpapers.repec.org/software/bocbocode/s459162.htm SCORE2 algorithms are available from https://www.phpc.cam.ac.uk/ceu/erfc/programs/

For manuscripts utilizing custom algorithms or software that are central to the research but not yet described in published literature, software must be made available to editors and reviewers. We strongly encourage code deposition in a community repository (e.g. GitHub). See the Nature Portfolio guidelines for submitting code & software for further information.

Data

Policy information about availability of data

All manuscripts must include a data availability statement. This statement should provide the following information, where applicable:

- Accession codes, unique identifiers, or web links for publicly available datasets
- A description of any restrictions on data availability
- For clinical datasets or third party data, please ensure that the statement adheres to our policy

To guarantee the confidentiality of personal and health information only the authors have had access to the data during the study in accordance with the relevant licence agreements. Access to the QResearch data is according to the information on the QResearch website (www.qresearch.org) and CPRD is available from www.cprd.com

Research involving human participants, their data, or biological material

Policy information about studies with human participants or human data. See also policy information about sex, gender (identity/presentation), and sexual orientation and race, ethnicity and racism.

Reporting on sex and gender	Analyses have been undertaken by biological sex (males/females)
Reporting on race, ethnicity, or other socially relevant groupings	We used self assigned ethnicity (as reported by the patient) for reporting.
Population characteristics	There were 9,976,306 people aged 18-84 in the QResearch English derivation cohort, 3,246,602 in the Qresearch English validation cohort and 3,542,007 in the CPRD validation cohort of the other three UK nations
Recruitment	We used data from two anonymised medical research databases derived from routinely collected NHS data
Ethics oversight	The Qresearch ethics approval is provided by the East Midlans Derby Research Ethics Committee [reference 18/EM/0400]. The CPRD ERAP approval reference is 20_000162

Note that full information on the approval of the study protocol must also be provided in the manuscript.

Field-specific reporting

Please select the one below that is the best fit for your research. If you are not sure, read the appropriate sections before making your selection.

☒ Life sciences ☐ Behavioural & social sciences ☐ Ecological, evolutionary & environmental sciences

For a reference copy of the document with all sections, see nature.com/documents/nr-reporting-summary-flat.pdf

Life sciences study design

All studies must disclose on these points even when the disclosure is negative.

Sample size	All available data on the database was used to maximise the available power.
Data exclusions	We excluded patients with pre-existing CVD, those prescribed statins and (for QResearch) those with a missing Townsend deprivation score since they often reprent temporary patients or incompletely registered with substantial missing data
Replication	We undertook two validation analyses in cohorts which had not been used for model development to determine the performance of the algorithm
Randomization	This is a prediction modeling study and not a clinical trial so randomisation was not part of the study design.
Blinding	This is a prediction modeling study and not a clinical trial so blinding was not part of the study design.

Reporting for specific materials, systems and methods

We require information from authors about some types of materials, experimental systems and methods used in many studies. Here, indicate whether each material, system or method listed is relevant to your study. If you are not sure if a list item applies to your research, read the appropriate section before selecting a response.

Materials & experimental systems

n/a	Involved in the study	
☒	☐	Antibodies
☒	☐	Eukaryotic cell lines
☒	☐	Palaeontology and archaeology
☒	☐	Animals and other organisms
☒	☐	Clinical data
☒	☐	Dual use research of concern
☒	☐	Plants

Methods

n/a	Involved in the study	
☒	☐	ChIP-seq
☒	☐	Flow cytometry
☒	☐	MRI-based neuroimaging

Plants

Seed stocks	n/a
Novel plant genotypes	n/a
Authentication	n/a
